# Skin Multi-Omics-Based Interactome Analysis: Integrating the Tissue and Mucus Exuded Layer for a Comprehensive Understanding of the Teleost Mucosa Functionality as Model of Study

**DOI:** 10.3389/fimmu.2020.613824

**Published:** 2021-02-04

**Authors:** Felipe E. Reyes-López, Antoni Ibarz, Borja Ordóñez-Grande, Eva Vallejos-Vidal, Karl B. Andree, Joan Carles Balasch, Laura Fernández-Alacid, Ignasi Sanahuja, Sergio Sánchez-Nuño, Joana P. Firmino, Leonardo Pavez, Javier Polo, Lluis Tort, Enric Gisbert

**Affiliations:** ^1^ Departament de Biologia Cel·lular, Fisiologia i Immunologia, Universitat de Autònoma de Barcelona (UAB), Bellatera, Spain; ^2^ Facultad de Medicina Veterinaria y Agronomía, Universidad de Las Américas, Providencia, Chile; ^3^ Consorcio Tecnológico de Sanidad Acuícola, Ictio Biotechnologies S.A., Santiago, Chile; ^4^ Departament de Biologia Cel·lular, Fisiologia i Immunologia, Universitat de Barcelona (UB), Barcelona, Spain; ^5^ Centro de Biotecnología Acuícola, Facultad de Química y Biología, Universidad de Santiago de Chile, Edificio de Investigación Eduardo Morales, Santiago, Chile; ^6^ IRTA-SCR, Aquaculture Program, Sant Carles de la Rápita, Spain; ^7^ PhD Program in Aquaculture, Universitat Autònoma de Barcelona, Bellaterra, Spain; ^8^ Instituto de Ciencias Naturales, Universidad de las Américas, Santiago, Chile; ^9^ APC Europe SL, Granollers, Spain

**Keywords:** microarrays, multi-omics analyses, functional networks, integrative analysis, immune response, histology, skin mucosa, functional diet

## Abstract

From a general structural perspective, a mucosal tissue is constituted by two main matrices: the tissue and the secreted mucus. Jointly, they fulfill a wide range of functions including the protection of the epithelial layer. In this study, we simultaneously analyzed the epithelial tissue and the secreted mucus response using a holistic interactome-based multi-omics approach. The effect of the gilthead sea bream (*Sparus aurata*) skin mucosa to a dietary inclusion of spray-dried porcine plasma (SDPP) was evaluated. The epithelial skin microarrays-based transcriptome data showed 194 differentially expressed genes, meanwhile the exuded mucus proteome analysis 35 differentially synthesized proteins. Separately, the skin transcripteractome revealed an expression profile that favored biological mechanisms associated to gene expression, biogenesis, vesicle function, protein transport and localization to the membrane. Mucus proteome showed an enhanced protective role with putatively higher antioxidant and antimicrobial properties. The integrated skin mucosa multi-interactome analysis evidenced the interrelationship and synergy between the metabolism and the exuded mucus functions improving specifically the tissue development, innate defenses, and environment recognition. Histologically, the skin increased in thickness and in number of mucous cells. A positive impact on animal performance, growth and feed efficiency was also registered. Collectively, the results suggest an intimate crosstalk between skin tissue and its exuded mucus in response to the nutritional stimulus (SDPP supplementation) that favors the stimulation of cell protein turnover and the activation of the exudation machinery in the skin mucosa. Thus, the multi-omics-based interactome analysis provides a comprehensive understanding of the biological context of response that takes place in a mucosal tissue. In perspective, this strategy is applicable for evaluating the effect of any experimental variable on any mucosal tissue functionality, including the benefits this assessment may provide on the study of the mammalian mucosa.

## Introduction

Skin is a stratified squamous epithelial surface strategically located at the interface with the external environment, where it has evolved to detect, integrate and respond to a diverse range of *stimuli* from the environment, including stressors and aggressions (1–3). Skin function is a crucial component for organism survival, acting like physical barrier as the outermost organ and requiring precise calibration of its responses with a high degree of local autonomy (4, 5). Described as the body’s largest organ, the vertebrate integument is a conserved organized structure consisting of the epidermis, dermis, and hypodermis (6, 7). Nonetheless, the skin of terrestrial and aquatic vertebrates has acquired differential specific adaptations because of its relationship with the environment. Whereas mammalian skin acquired dead keratinized cell layers, hair follicles, sweat glands but loosed mucus production capacity (8), the teleost skin presents mucous glands, which produce antifungal and antibacterial substances, and it also serves as an osmotic barrier (9). According to these characteristics, teleost mucosal surfaces may closely resemble type I mucosal surfaces of mammals, represented by the intestine, the respiratory tract and the uterus, exerting similar physiological (10) and immunological functions (11, 12). For instance, from an evolutionary point of view, regardless of their phylogenetic origin and tissue localization, mucosal immunoglobulins operate under the guidance of primordially conserved principles from fish to mammals (13). Moreover, the systematic exploration of fish skin models have been proposed as biologically, clinically and technologically relevant, opening interesting new opportunities for dermatological research (14). Regarding fish skin, it is well assumed that this interface tissue also acts as a multifunctional organ, playing roles in protection, communication, sensory perception, locomotion, respiration, ion regulation, excretion, and thermal regulation (15).

Research on mucosal tissues mainly tackled unique and evolved immune mechanisms of defense in mammals as well as in fish. The epithelial structure, including impermeable tight junctions, goblet cells distribution and density, or the different mucosa-associated lymphoid tissues (MALTs) are of major interest to understand mucosal properties in mammals (16, 17) as well as in fish species (13, 15, 18). The maintenance of these mucosal tissues in healthy conditions is complex and relies on a delicate balance between the diet, the commensal microbiota and the mucosa itself, including epithelia and the overlying mucus layer. Numerous studies have described the benefits of an adequate diet or the dietary additives to enhance human and animal condition and welfare, with special attention on gut health. However, efforts to intensify animal production of valuable species can lead to increased stress, limited growth performance and poor welfare; thus, the research for nutritional strategies focused on “functional feeds” is a priority task (19–22).

Spray-dried porcine plasma (SDPP) is an abattoir by-product obtained from animal blood after exclusion of cells, and subjected to concentration and spray drying (23). It has been widely used as a safe and high-quality feed ingredient for livestock, especially at the time of weaning because this ingredient promotes feed intake, somatic growth and reduces stress, as well as morbidity and mortality (23–26). Furthermore, several proteins with distinct functions have been found in SDPP such as immunoglobulins, albumin, growth factors and biologically active peptides, which mediated anti-inflammatory effects (27–29). Regarding aquatic species, several studies have reported that SDPP enhances growth in rainbow trout (*Oncorhynchus mykiss*) (30), gilthead seabream (*Sparus aurata*) (31) and Nile tilapia (*Oreochromis niloticus*) (32). These results may be attributed to its high digestibility, the improvement of feed intake and feed efficiency, as well as its content in growth promoting factors. Recently, the dietary inclusion of SDPP has shown to enhance innate immunity and antioxidant enzyme activities in gilthead seabream (31). These results are in agreement with gained evidence in higher vertebrates, which hypothesizes that SDPP protects the organism *via* the immune system or directly acting against pathogens (27).

Multi-omics approaches pursue the integration of different biological entities to understand their interrelation and the functioning of larger systems, and serve to identify new biomarkers in specific tissues (33–35). In general, all experimental ‘omics’ approaches can be considered to share some major features in contrast to traditional procedures. However, single omics analysis does not always provide enough information to understand the behavior and responsiveness of a cellular system. Therefore, a combination of multiple omics analyses, the so-called multi-omics approach, is required to acquire a precise picture of living organisms (36). ‘Omics’ are high-throughput, holistic, top-down methodologies and data-driven, which also attempt to understand the cell metabolism like an ‘integrated system’, rather than as mere collections of different parts by using information of the relationships between many measured molecular species (36). Several popular ‘omics’ platforms in tissue biological systems include transcriptomics, which measures mRNA transcript levels; proteomics, the set of proteins expressed by an organism, tissue, or cell; metabolomics, which determines abundance of small cellular metabolites; and interactomics, which integrates the whole set of molecular interactions in cells. To date, information regarding cellular metabolism has been acquired through application of individual ‘omics’ approaches (37), but scarce studies tackled on multi-omics data to analyze tissue functionality and none on mucosal tissues, including epidermal/epithelial cells and its mucus exudation.

The present study proposes a multi-omic analysis for the sustained dietary supplementation impacts of a functional diet including 3% of SDPP on fish skin mucosa. The fish model selected was the gilthead sea bream due to its well-known physiology and its high economical value. To propose an adequate description of the mucosal skin response, we combined several molecular biological disciplines that measure the entirety of biomolecules differentially expressed by means of skin transcriptomic analyses and the modification on mucus layer exudation by the analysis of the mucus proteome. Histological analyses for skin structure and mucous cell density, growth performance and feed efficiency parameters were also performed. Collectively, the results suggest the whole-mucosal interactome as a useful strategy for representing the beneficial effects of functional diets. Prospectively, this methodology arises as a promising alternative for the applicability of treatments upon the mammalian type I mucosal surfaces.

## Materials and Methods

### Diets

To assay how a functional diet would benefit skin mucosa functionality two diets were formulated as follows: a control diet (Diet C), equivalent to commercial diet containing 51% crude protein, 17% crude fat and 20.6 MJ/kg gross energy that fulfill the nutritional requirements of juvenile sea bream. Based on this basal formulation, another diet named Diet SDPP was manufactured where Fishmeal LT70 was substituted by 3% SDPP (Apetein GS, APC Europe SL, Granollers, Spain; **Table 1**). Diets were manufactured by Sparos Lda (Portugal). In brief, main ingredients were ground (<250 μm) in a micropulverizer hammer mill (Hosokawa Micron). Powder ingredients and oils were then mixed according to the target formulation in a paddle mixer (RM90; Mainca). All diets were manufactured by temperature-controlled extrusion (pellet sizes: 0.8 and 1.5 mm) by means of a low-shear extruder (P55; Italplast). Upon extrusion, all feed batches were dried in a convection oven (OP 750-UF; LTE Scientific) for 4 h at 45°C.

**Table 1:** Ingredient list and proximate composition on dry weight basis (%) of experimental diets.

Ingredients	Diets	
Ingredients (%)	Control	SDPP
Fishmeal LT 70	36.90	33.35
Fishmeal 60	12.50	12.50
CPSP 90	4.00	4.00
Squid meal	6.00	6.00
Appetein GS		3.00
Wheat Gluten	7.60	7.60
Soybean meal 48 (micronized)	7.00	7.00
Wheat meal	7.70	7.70
Pea starch	4.50	4.80
Fish oil	11.20	11.45
Vitamin and Mineral Premix PV01	1.00	1.00
Choline chloride	0.10	0.10
Soy lecithin	0.50	0.50
Binder (guar gum)	1.00	1.00
**Total**	**100.00**	**100.00**
**Proximate composition basis**	**CTRL**	**SDPP**
Crude protein (%)	51.09	51.11
Crude fat (%)	17.17	17.16
Fiber (%)	0.51	0.51
Ash (%)	11.75	11.38
Gross Energy (MJ/kg)*	20.56	20.69
**Amino acid composition (as feed basis)**		
Histidine	1.05	1.08
Isoleucine	2.00	2.02
Leucine	3.76	3.85
Lysine	3.52	3.60
Threonine	2.35	2.40
Tryptophan	0.54	0.56
Valine	2.32	2.39
Methionine + Cysteine	1.82	1.84
Phenylalanine + Tyrosine	4.32	4.42
Taurine	0.16	0.15

*Gross energy content was estimated by using the following: total carbohydrate = 17.2 J/kg, fat = 39.5 J/kg and protein = 23.5 J/kg.

### Fish and Experimental Design

Gilthead seabream fry (average body size 9.5 g) were obtained from a commercial hatchery (Piscimar SL, Andromeda Group, Spain) and transported by road to IRTA-Sant Carles de la Rapita research facilities (Sant Carles de la Ràpita, Spain), where they were acclimated in two 2000-L tanks for two weeks. After their acclimation, all fish were anesthetized (tricaine methanesulfonate [MS-222], 150 mg/L) and individually weighed for initial body weight (BWi)

and measured for standard length (SLi) to the nearest 0.1 g and 1 mm, respectively, and then distributed into eight 500-L cyclindroconical tanks at a density of 50 fish per tank (4 tanks/replicates per diet).

Fish (BWi = 10.6 ± 0.1 g, n = 400, mean ± standard deviation, SD) were fed for 95 days with both experimental diets by means of automatic feeders (ARVO-TEC T Drum 2000; Arvotec, Huutokosk, Finland) at the rate of 2.5% of the stocked biomass, which approached apparent satiation. Feed ration was evenly distributed in seven meals per day from 08:00 to 18:00 h. Fish were regularly sampled at a monthly basis in order to evaluate their growth in BW and adjust the feeding ratio to stocked biomass. During the trial, water temperature and pH (pH meter 507; Crison Instruments, Barcelona, Spain), salinity (MASTER-20T; ATAGO Co., Ltd., Tokyo, Japan), and dissolved oxygen (OXI330; Crison Instruments) were 22.1 ± 0.4°C, 7.0 ± 0.01, 36 mg/L, and 7.2 ± 0.3 mg/L (mean ± SD), respectively. Water flow rate in experimental tanks was maintained at approximately 9.0–10.1 liter/min *via* a recirculation system (IRTAmar^®^; Spain) that maintained adequate water quality (total ammonia and nitrite were ≤0.15 and 0.6 mg/L, respectively) through UV, biological, and mechanical filtration. Photoperiod followed natural changes according to the season of the year (November to February; 40°37′41″ N).

At the end of the trial, fish were anaesthetized as previously described; mucus was gently scrapped off from the skin surface (n = 15 per diet) using a sterile glass slide avoiding blood, urine and feces during collection and transferred into 2-ml Eppendorf tubes and stored at -80 °C as is described in Fernández-Alacid et al. (38). All fish in experimental tanks were measured for final BW and SL as indicated. Then, 10 fish per tank were sacrificed with an overdose of MS-222 (200 mg/L) for tissue sampling purposes. Skin samples (1 cm^2^ of anterior dorsal body region) were dissected and immediately transferred into RNAlater (Ambion^®^), fixed overnight and then frozen at -80°C until further RNA extraction. Fish growth and feed utilization from experimental groups was evaluated by means of the following indices: Fulton’s condition factor (K) = (BW/SL^3^) × 100; specific growth rate in BW (SGR, %) = ((ln BWf −ln BWi) × 100)/time (d) and feed conversion ratio (FCR, g/g) = FI/(Bf −Bi), where FI was the total feed intake during the experimental period (g) and, Bi and Bf were the initial and final biomass (g), respectively.

All animal experimental procedures were conducted in compliance with the experimental research protocol approved by the Committee of Ethics and Animal Experimentation of the Institut de Recerca i Tecnologia Agroalimentàries and in accordance with the Guidelines of the European Union Council (86/609/EU) for the use of laboratory animals.

### Histological Organization of the Skin

For histological purposes, 1 cm^2^ of the skin from the dorsal anterior region of the body from 16 fish per dietary treatment was dissected (n= 4 fish per tank) and fixed in 4% buffered formaldehyde (pH = 7.4), dehydrated in a graded series of ethanol, cleared with xylene, embedded in paraffin and cut in serial sections (3–5 μm thick). Sections were stained with hematoxylin-esosin (HE) for general histological descriptions, whereas slides were stained with Periodic Acid Schiff for goblet cell identification (neutral mucins produced by mucous cells stain in magenta). All sections were observed under a light microscope (Leica DM LB; Leica Microsystems) and photographed (Olympus DP70 Digital Camera; Olympus Imaging Europa GmbH). Digital images (600 dpi) were processed and analyzed using an image analysis software package (ANALYSIS; Soft Imaging Systems GmbH). Measurements of the thickness of different skin regions (epidermis and dermis), as well as mucous cell number (full and empty) were based on the analysis of three to five randomly chosen fields from the skin per fish. The number of epidermal mucous cells was expressed over a length of 100 μm.

### Transcriptional Analysis

Total RNA was extracted individually from fish skin using QIAGEN RNeasy^®^ Mini Kit following the manufacturer’s recommendations. The total RNA concentration was quantified using a NanoDrop ND-2000 (Thermo Scientific) and RNA integrity and quality checked with the Experion (Automated Electrophoresis Station, Bio-Rad) using the Experion Standard Sens RNA chip (Bio-Rad). Only the samples with an RNA integrity number (RIN) > 8.0 were chosen for further analysis. Transcriptional analysis was carried out using the AquaGenomic *Sparus aurata* oligonucleotide microarray (SAQ) platform (39). The complete information on this platform and our data is available through the public repository Gene Expression Omnibus (GEO) (accession numbers GPL13442 and GSE162501, respectively) at the United States National Center for Biotechnology Information (NCBI). A transcriptomic analysis was conducted to determine differences at the expression level between control and SDPP groups at the end of feeding trial (95 days). For each experimental group (control; SDPP group) total RNA samples were pooled (n = 3 pools each group; n = 4 fish each pool, n = 1 fish taken at random from each tank for each pool) using the same final concentration (133 ng/µL each pool). One-color microarray was carried out according to the manufacturer’s protocols. Briefly, 200 ng of total RNA was reversed transcribed along with spike-in (Agilent One-Color RNA Spike-In kit, Agilent Technologies, United States). The solution was then used as template for Cyanine-3 (Cy3) labeled cRNA synthesis and amplification with the Quick Amp Labeling kit. cRNA samples were purified using the RNeasy micro kit (Qiagen) according to manufacturer’s instructions. Dye incorporation and cRNA yield were checked with the NanoDrop ND-2000 Spectrophotometer. 1.5 mg of Cy3-labeled cRNA with specific activity of >6.0 pmol Cy3/mg cRNA was then fragmented at 60°C for 30 min, and the samples were then mixed with hybridization buffer and hybridized to the array (ID 025603, Agilent Technologies) at 65°C for 17 h, using the Gene expression hybridization kit. Washes were conducted as recommended by the manufacturer, using gene expression wash buffers and a stabilization and drying solution (Agilent Technologies). Microarray slides were scanned with Agilent Technologies Scanner model G2505B. Spot intensities and other quality control features were extracted with Agilent’s Feature Extraction software version 10.4.0.0 (Agilent Technologies). Quality reports were checked for each array. The extracted raw data were imported and analyzed with GeneSpring (version 14.5 GX software, Agilent Technologies). The 75% percentile normalization was used to standardize the arrays for comparisons, and data were filtered by expression. The differential expressed genes (DEGs) were obtained from a gene-level differential expression analysis. Expression values with a p-value < 0.05 were considered statistically significant. The DEGs were grouped according to its fold-change value (p-value < 0.05) and represented using the GraphPad software v7.0 for Windows. The Principal Component Analysis (PCA) was carried out using GeneSpring software (Agilent), four eigenvectors were calculated to describe the aggrupation of the control and SDPP groups in a 3D plot. The gene expression values (log2-expression ratios) were represented by a hierarchical clustering heatmap analysis using MeV software (v4.0), with Pearson distance and average linkage as it was described before (40).

#### Gene Ontology and Pathway Enrichment Transcriptome Analysis

In order to classify the DEGs (both up- and down-regulated) according to its functional annotation, genes were imported into the web-tool Protein ANalysis Through Evolutionary Relationships (PANTHER) classification system (version 13.0) (41). This web resource allows understanding the biological meaning behind large list of DEGs based on their GO classification. For the enrichment analysis, the major over-represented GO were chosen according to a p-value < 0.05 criteria (biological processes; cellular component). The biological interpretation of the DEGs obtained was complemented using the free access databases GeneCards (www.genecards.org) (42) and UniProt (www.uniprot.org) (43).

### Proteomic Analysis of Exuded Mucus

#### Protein Extraction and Two-Dimensional Electrophoresis Separation

Mucus samples for two-dimensional electrophoresis (2-D PAGE) protocols were solubilized in equal volume of ice-cold lysis buffer (7 M urea; 2 M thiourea, 2% w/v CHAPS and 1% protease inhibitor mixture) and centrifuged at 20,000 *g* for 15 min at 4°C, whereas the resultant supernatant was aliquoted avoiding pellet resuspension and surface lipid layer. The supernatants obtained were submitted to a clean-up procedure (ReadyPrep 2-d clean-up kit, Bio-Rad) in order to enhance protein extraction as described in Sanahuja and Ibarz (44). The proteome map of soluble epidermal mucus proteins was obtained by 2D-electrophoresis. Protein concentration was determined by Bradford assay with bovine serum albumin as standard (Bio-Rad).

Pools of three mucus samples were prepared in order to obtain 450 µg of protein dissolved in 450 µL of rehydration buffer containing 7M urea, 2M thiourea, 2% w/v CHAPS, and 0.5% v/v IPG buffer, 80 mM DTT and 0.002% bromophenol blue. Five samples of skin mucus protein extract from each dietary condition (Control and SDPP diets) were loaded onto 24 cm, pH 3–10 NL IPG strips (GE Healthcare, Madrid, Spain). Isoelectric-focusing was performed using an IPGhor instrument (Amersham Biosciences), following the manufacturer’s instructions (active rehydratation at 50 V for 12 h followed by linear gradient from 500 to 8000 V until 48,000 V/h). The focused strips were equilibrated in two steps as follows: 15 min with equilibration buffer I (65 mM DTT, 50 mM Tris-HCl, 6 M urea, 30% glycerol, 2% SDS, bromophenol blue) and then, 15 min with equilibration buffer II (135 mM iodoacetamide, 50 mM Tris-HCl, 6 M urea, 30% glycerol, 2% SDS, bromophenol blue). Equilibrated strips were set directly onto 12.5% polyacrylamide gels, sealed with 0.5% w/v agarose, and separated at a constant voltage of 50 V for 30 min followed by 200 V for about 6 h, until the blue dye reached the bottom of an Ettan DALT II system (Ammersham Biosciences, Stockholm, Sweden). Proteins were fixed for 1 h in methanol: acetic acid 40:10 and stained overnight using colloidal Coomassie blue G-250. Gel staining was removed by consecutive washing steps with distilled water until the best visualization was achieved.

#### Gel Image Analysis and Protein Digestion

Coomassie blue stained gels were scanned in a calibrated Imagescanner (Bio-Rad, Spain) and digital images captured using Quantity-One software (Bio-Rad). The images were saved as uncompressed TIFF files. Gel images were analyzed using the software package ImageMaster 2D, version 6.01 (GE Healthcare, Spain). Proteins were detected using the automated routine of ImageMaster 2.0 software, combined with manual editing when necessary to remove artefacts. The background was removed and normalized volumes were calculated as follows: the volume of each protein spot was divided by the total volume of all the protein spots included in the analysis. Normalized protein spot values were used to select the 300 most abundant proteins in each condition to be further analyzed for their differential expression. The obtained protein spots with differential expression, henceforth differential expressed spots (DESs) were manually cut from the gel and in-gel tryptic digestion was performed in an InvestigatorTM Progest (Genomic Solution) automatic protein digestion system as it was detailed for fish mucus samples in Sanahuja and Ibarz (44).

#### LC-MS/MS Analysis and Database Search

Dried-down peptide mixtures were analyzed in a nanoAcquity liquid chromatographer (Waters) coupled to a LTQ-Orbitrap Velos (Thermo Scientific) mass spectrometer. Tryptic digests were resuspended in 1% FA solution and an aliquot was injected for chromatographic separation. Peptides were trapped on a Symmetry C18TM trap column (5 µm 180 µm x 20mm, Waters), and separated using a C18 reverse phase capillary column (ACQUITY UPLC M-Class Peptide BEH column; 130 Å, 1.7µm, 75 µm x 250mm, Waters). The gradient used for the elution of the peptides was 1 to 40% B in 20 min, followed by gradient from 40 to 60% during 5 min (A:0.1% FA; B: 100% CAN, 0.1% FA), with a flow rate of 250 nl/min. Eluted peptides were subjected to electrospray ionization in an emitter needle (PicoTipTM, New Objective) with an applied voltage of 2,000 V. Peptide masses (m/z 300–1,700) were analyzed in data dependent mode where a full Scan MS was acquired in the Orbitrap with a resolution of 60,000 FWHM at 400 m/z. Up to the 10th most abundant peptides (minimum intensity of 500 counts) were selected from each MS scan and then fragmented in the linear ion trap using CID (38% normalized collision energy) with helium as the collision gas. The scan time settings were: Full MS: 250 ms (1 microscan) and MSn: 120 ms. Generated.raw data files were collected with Thermo Xcalibur (v.2.2).

Files obtained from mass spectrometry analyses were used to search against the public database Uniprot Actinopterygii (v.23/3/17). A database containing common laboratory contaminant proteins was added to this database. The software used as Thermo Proteome Discoverer (v1.4.1.14) with Sequest HT as the search engine. The following search parameters were applied: two missed cleavage sites as well as fixed and variable modifications; carbamidomethyl of cysteine and oxidation of methionine, respectively. Peptide tolerance was 10 ppm and 0.6 Da for MS and MS/MS spectra, respectively. Both target and decoy databases were searched in order to obtain a false discovery rate (FDR), and thus, estimate the number of incorrect peptide-spectrum matches that exceeded a given threshold. The results were filtered so only proteins identified with at least two high confidence (FDR >1%) peptides were included in the lists. To sort the search results, proteins were ranked by a first criterion of the higher Score together with and a second criterion of the higher number of Sequence Coverage and Peptides matched. The principal component analysis (PCA) was used to check the quality of the data from each replicate and identify the subsets of samples that are associated with the two different groups under study. The protein intensity values (log2-expression ratios) were represented by a hierarchical clustering heatmap analysis using MeV software (v4.0), with Pearson distance and average linkage.

### Functional Network Analyses: Interactomes

The complete map of interactions that can occur in a living organism (interactome) was obtained from the DEGs obtained in the microarrays-based transcriptomics analysis (transcripteractome), from the DESs (proteinteractome) and the functional integrated network for the transcriptomic and proteomic outcomes both together (multinteractomics). For this purpose, the Search Tool for the Retrieval of Interacting Genes (STRING) public repository version 10.0 (https://string-db.org) was used (45). Protein-protein interaction (PPI) network for the differentially expressed genes was conducted with a high-confidence interaction score (0.9). The mechanisms of response in which DEGs, DESs and both omics outcome together are involved, were obtained from a comparative analysis based on *Homo sapiens* as a reference organism in order to extract the maximum information currently available. Thus, an orthologue *H. sapiens* Entrez Gene ID was assigned based on sequence homology. Briefly, we selected the best tBlastX (NCBI) hit between the entire set of DEGs and DESs query sequence for *S. aurata* and the human transcriptome database. We only consider those matches with at least E value ≤ 1E-10. The Uniprot (43) and Genecards databases (42) were used to confirm match of the gene acronym tag between both species. The detailed list of human orthologues is available on **Table S9**. Gene ontology (GO) pathway enrichment analysis (biological processes; cellular component) was also performed for the DEGs, DESs, and both entities together (DEGs+DESs) by STRING using a Fisher’s exact test followed by a correction for multiple testing (46, 47). A p-value < 0.05 was considered as significant. The GO terms obtained were then identified in the ancestor GO chart using the QuickGO web-tool (https://www.ebi.ac.uk/QuickGO/) (48). The GO chart for each GO obtained from the enrichment analysis were then mapped in a single chart in order to identify those less redundant GO terms and thus propose more stringent GO terms associated to the mechanism of response for the SDPP-fed fish. The potential interaction between the GO found into the functional networks was estimated based on an integrative cluster analysis. To do it, those more stringent statistically significant GO obtained from the enrichment analysis were assigned to each one of the nodes represented in the functional network. The nodes classified in different clusters according to their functionality were represented by integrative ameboid graphics using Adobe Photoshop version CC2018.

### Statistical Analysis

Data on growth performance, feed conversion and skin morpohometrics were compared by means of a t-test. Regarding, microarray data, an unpaired t-test) was conducted using the GeneSpring software GX 14.5 to detect DEGs (p < 0.05) between the control and SDPP groups. Mucus differential synthesized proteins (DESs) that were found to vary in their abundance between the control and SDPP diets were analyzed for significance using a t-test. The Shapiro-Wilk test was first used to ensure the normal distribution of the data, while the uniformity of the variances was determined by Levene’s test. The DEGs and DESs fold-change graphs were represented with GraphPad software version 7.0. The PCA analyses for DEGs and DESs were obtained from GeneSpring software version 14.5 (Agilent Technologies) and Analyse-it Software versión 5.4, respectively.

### Ethics Statement

Animal experimental procedures were conducted in compliance with the research protocol approved by the IRTA’s Committee of Ethics and Animal Experimentation and in accordance with the Guidelines of the European Union Council (86/609/EU) for the use of laboratory animals.

## Results

### Profiling of the Skin Transcriptome and the Mucus Proteome

**Figure 1:**
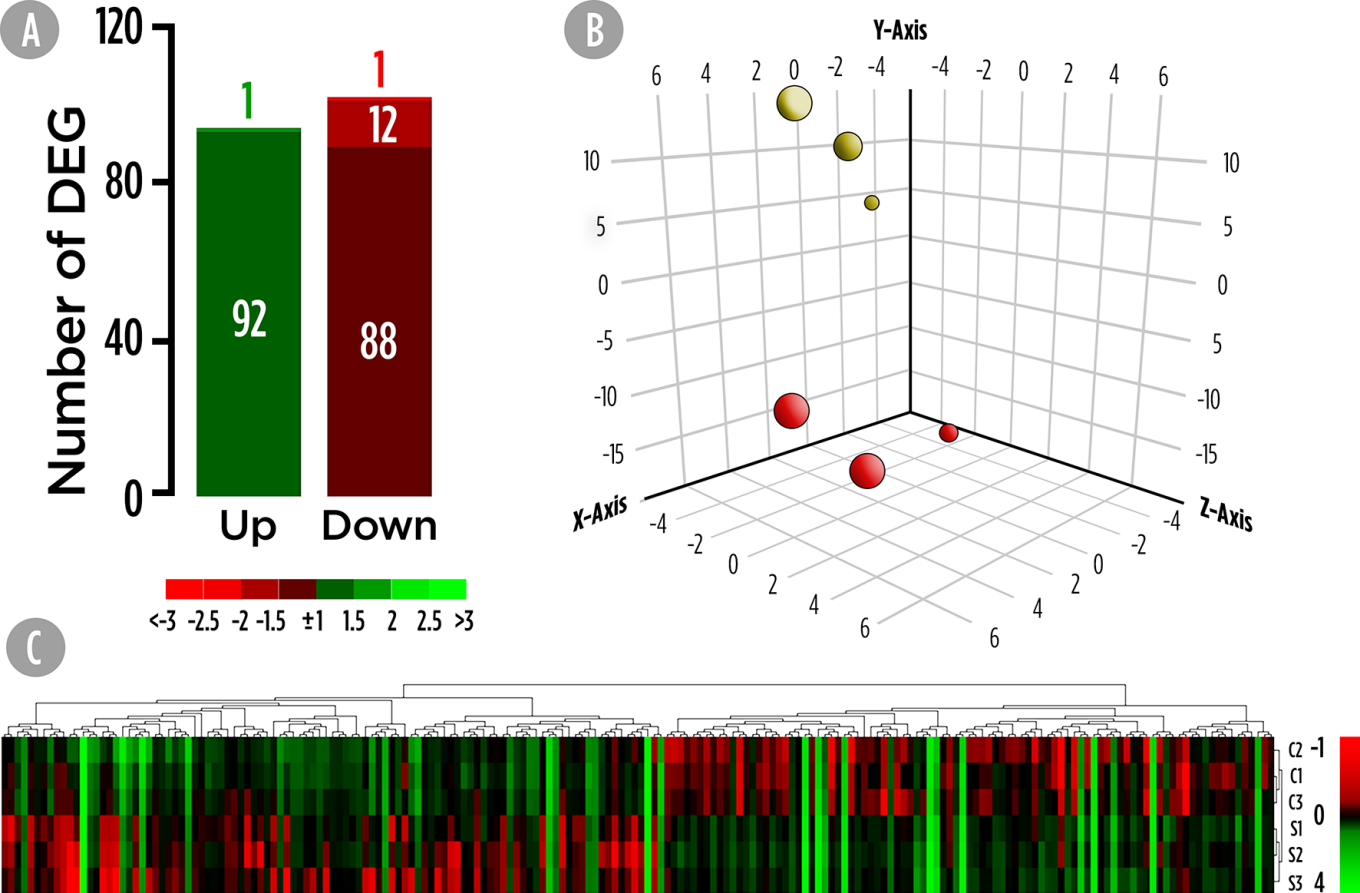
Skin mucus microrrays-based transcriptomic analysis for gilthead sea bream fed with SDPP supplemented diet. **(A)** Number of total differential expressed genes (DEGs). The green (upregulation) and red (downregulation) color scheme indicates the gene modulation according to its fold-change magnitude interval. **(B)** Principal component analysis (PCA). Control (red spheres) and SDPP groups (yellow spheres) are represented. **(C)** Hierarchical clustering heatmap representing the 194 DEGs. The normalized intensity values (log2) obtained for each microarray analyzed for control (C1, C2, and C3) and SDPP group (S1, S2, and S3) are shown.

Transcriptional analysis of the skin was carried out using the *S. aurata* oligonucleotide custom microarray and results are shown in **Figure 1**. A total of 194 DEGs were found in the skin of fish fed the SDPP diet (**Figure 1A**). Among them, 121 DEGs were annotated, whereas 73 were unknown genes (**Table S1**). Among them, 93 DEGs were up-regulated and 101 down-regulated. Results from the PCA divided the dataset into three principal components and revealed a clear differential gene expression pattern among the control and SDPP groups (**Figure 1B**). When representing DEGs from both experimental groups using a hierarchical clustering heatmap, we found a clear differential expression profile with a clear grouping of DEGs of fish fed with the control diet (**Figure 1C**; top half of the panel) compared to those fed the SDPP diet (**Figure 1C**; bottom half).

**Figure 2:**
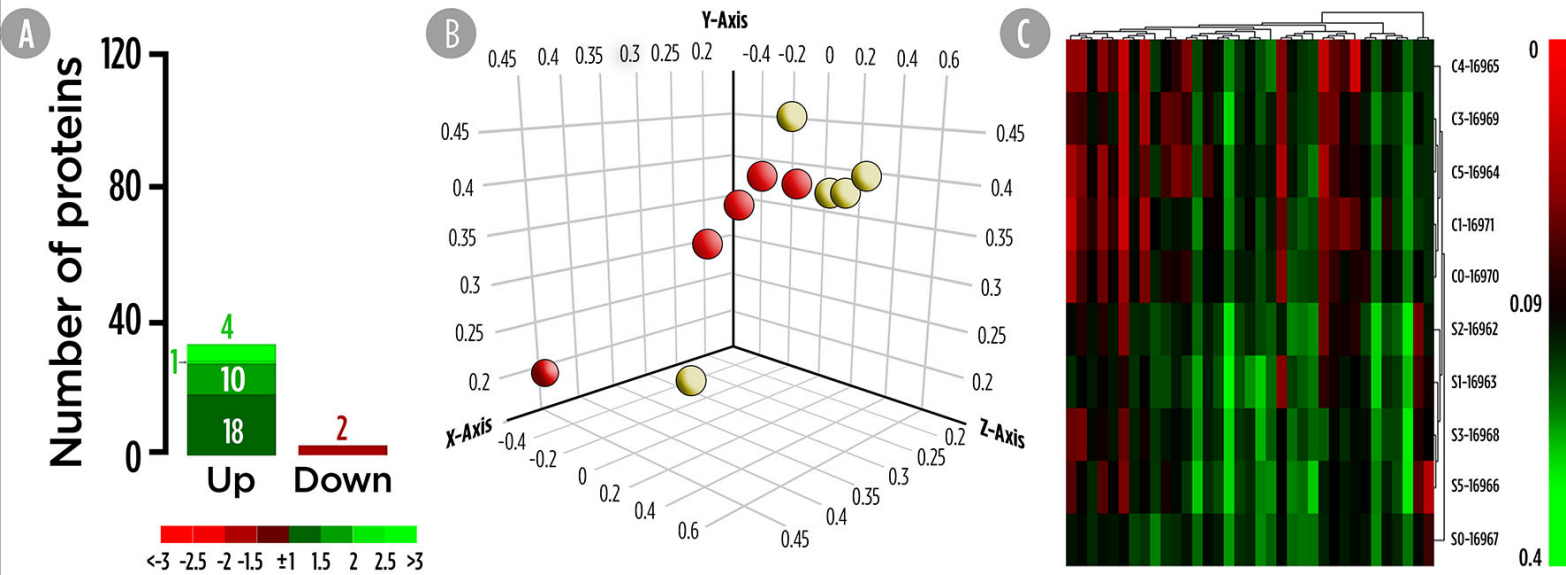
Skin mucus 2-Dimensional electrophoresis separation-based proteomic analysis for gilthead sea bream fed with SDPP supplemented diet. **(A)** Number of total differential expressed spots (DESs). The green (upregulation) and red (downregulation) color scheme indicates the protein modulation according to its fold-change magnitude interval. **(B)** Principal component analysis (PCA). Control (red spheres) and SDPP groups (yellow spheres) are represented. **(C)** Hierarchical clustering heatmap representing the 35 DESs. The results obtained for each 2-D electrophoresis separation for control (C0, C2, C3, and C4) and SDPP group (S0, S1, S2, S3, and S4) are shown.

High-resolution 2D maps of epidermal mucus proteomes were obtained for each individual sample by a combination of broad range, 3–10NL IPG strips with large format SDS gels. In brief, 950 protein spots were detected in the mucus proteome of all samples after 2DE-gel staining. In primary matched sets, a representative master gel was obtained for the Control diet (**Figure S1**) and the 300 spots with higher normalized intensity were further analyzed for their differently synthesis between both experimental groups. A total of 35 proteins, whose abundance were significantly changed, were considered as DESs since they accomplished the criterion over the 2-fold spot intensity difference. Importantly, none of the significant proteins that changed matched with the identified DEGs. A total of 33 proteins were up-regulated (**Figure 2A**). The PCA analysis for DESs suggested a differential protein synthesis pattern between the skin mucus of gilthead seabream fed the control and SDPP diets (**Figure 2B**). This result was confirmed by the hierarchical clustering heatmap, showing a clear differential synthesis profile between both experimental groups; thus, grouping the DESs profile of the skin mucus proteome from fish fed with the control diet (**Figure 2C**; top half of the panel) compared to that of fish fed the SDPP diet (**Figure 2C**; bottom half).

### Transcripteractome: The Skin Transcriptomics Functional Network

**Figure 3:**
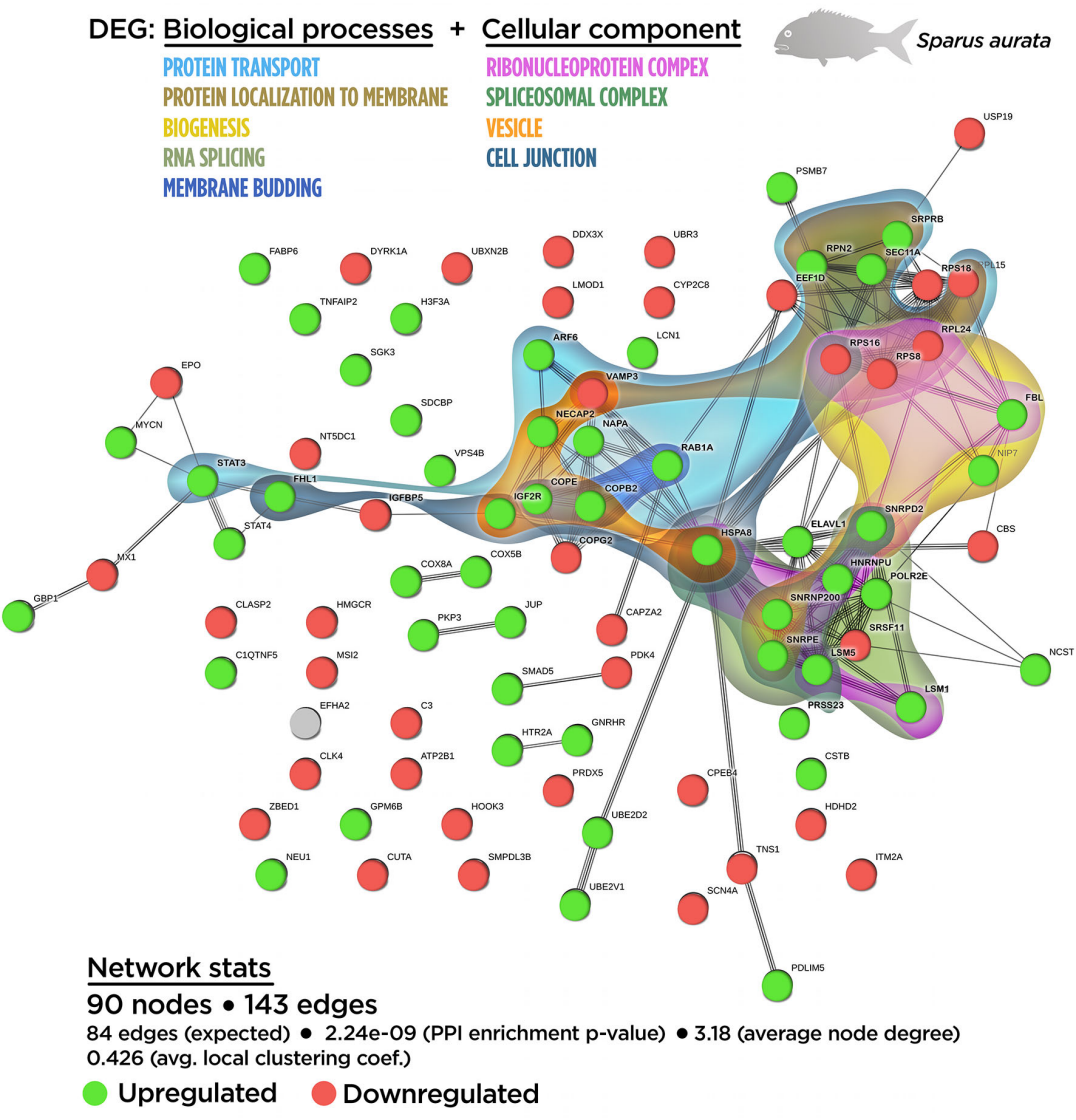
Integrative ameboid graphic for the biological processes and cellular components obtained from the skin transcripteractome analysis in gilthead sea bream fed with SDPP supplemented diet. Each node represents one differential expressed gene (DEG) obtained from the skin transcriptomic analysis. The DEG modulatory profile is represented with green (upregulated) and red (downregulated) into each node. The integrative cluster analysis groups the DEGs classified into the GO biological process or cellular component enrichment analysis. The set of these GO enrichment pathways are represented with different colors (bottom). The functional network statistics (Network stats) details are indicated (bottom).

A functional network analysis was carried out based on the DEGs obtained from the skin tissue transcriptional analysis from fish fed with the SDPP diet. From the 121 annotated DEGs, a functional association was registered between 90 DEGs, (74.4% of the total annotated DEGs) with the generation of 143 edges between them (**Figure S2**). In particular, 47 DEGs belonged to the central core interaction network (31 up-regulated DGEs; 16 down-regulated DEGs), while eight DEGs (seven up-regulated DEGs and one down-regulated DEGs) were more isolated. On the other hand, 35 DEGs (13 up-regulated DGEs; 22 down-regulated DEGs) showed no interaction with the main functional network obtained (**Figure S2**). The gene ontology (GO) pathway enrichment analysis revealed that the dietary inclusion of SDPP induced sustained changes in several biological processes including “RNA splicing” (GO.0008380) and “mRNA processing” (GO.0006397), “ribonucleoprotein complex biogenesis” (GO.0022613) and “ribosome biogenesis” (GO.0042254), “intracellular protein transport” (GO.0006886), and “protein localization to membrane” (GO.0072657) (**Figure S2**). Biological processes linked to “membrane budding” (GO.0006900) and “cellular catabolic process” (GO.0044248) were also detected. The full list of the GO biological process enrichment analysis is on **Table S2**. The complexity of the interaction between the biological processes was assessed through an integrative cluster analysis (**Figure 3**) where the sharing nodes of DEGs were considered as evidences of interacting network within biological processes.

In order to determine the functional network upon cellular structures, compartments, or macromolecular complexes, DEGs were classified according to their GO cellular component (**Figure S3; Table S3**). According to the GO terms for biological process enrichment analysis, the cellular component enrichment determined the presence of macromolecular complexes associated with splicing (GO.0097525 “spliceosomal snRNP complex”; GO.0005681 “spliceosomal complex”) and biogenesis (GO.0030529 “ribonucleoprotein complex”), whereas GO terms associated with vesicle compartment (GO.0030662 “coated vesicle membrane”; GO.0030135 “coated vesicle”) were also identified. Importantly, GO terms related to cell junction (GO.0070161 “anchoring junction”; GO.0005925 “focal adhesion”) were found, suggesting the effect of SDPP upon classical cellular structures for the maintenance epithelial tissue integrity.

The relationship between the cellular component GO terms was evaluated by an integrative cluster analysis. According to the data obtained for biological processes GO terms, the transcripteractome showed a strong association between spliceosomal complex (six up-regulated DEGs; zero down-regulated DEGs) and the ribonucleoprotein complex (seven up-regulated DEGs; three down-regulated DEGs) (**Figure S3**). Taking collectively both integrative clusters analyses of biological processes and cellular component for DEGs, a strong relationship was found between protein transport, vesicle compartment, protein localization to membrane, cell junction structures, and membrane budding (**Figure 3**).

### Proteinteractome: The Skin Mucus Proteomic Functional Network

The mucus proteome analysis determined the relative abundance for 35 DESs on the exuded skin matrix in fish fed the SDPP diet. Details on protein identification are supplied in **Table S4** providing the protein identity, gene symbol, fold change, the theoretical/observed MW and pI, together with the accession number, identified peptides, score, sequence coverage, species of identification and protein code by UniProtKB. From the total DESs, only two of them were down-regulated meanwhile the other 33 showed a clear higher relative abundance on the mucus of fish fed the SDPP diet compared to the control group (**Figure S4**). According to their function in epidermal mucus, DESs were classified as structural-, metabolic- or defense-related proteins (**Figure S4**). Among the DESs with protective-related roles, two groups of up-regulated proteins were found: proteins with chaperone activity and proteins with enzymatic defensive activities including proteasomal and esterase activity. Four identified proteins were associated with cell redox activity: the protein disulfide-isomerase related to protein disulfide bonds formation, and three enzymes related to glutathione biosynthesis, which participate in the synthesis of cysteine, glutamate and serine, respectively. Together with glutathione biosynthesis, a miscellaneous group of metabolic proteins and enzymes were up-regulated on the mucus of fish fed the SDPP diet. These results were in agreement to the described increased skin metabolic activity revealed by the transcripteractome. The third group of proteins belonged to the structural function of epidermal mucus, most of them participating in the process of mucus exudation. Thus, ten up-regulated proteins were identified as different keratin types (I and II). However, six of these keratin forms were located with a markedly lower molecular weight than expected (spots 14, 20, 22, 28, 31 and 32) and they could be described as “keratin fragments” resulting from own mucus enzymatic activity. Within the rest of structural proteins, two additional groups were proposed. The first one related to the cell exocytosis process, which includes up-regulated actin forms and cell motility-related proteins. The second one group of proteins related to cytoskeleton organization were also up-regulated except for a catenin form, a protein belonging to cadherin cell junction complex, which was down-regulated.

At the proteome functional network, 25 DESs were represented, including the 71.4% of the total 35 DESs (**Figure S5**). Among them, 22 DESs (21 up-regulated DESs; one down-regulated DESs), whereas they interacted each other totalizing 65 edges in the functional network. Only three DESs (one up-regulated DESs; one down-regulated DESs) showed no interaction with the functional network (**Figure S5**). The GO enrichment analysis showed that fish fed with the SDPP diet presented a modulatory effect on several biological processes, including “epidermal cell differentiation” (GO.0009913) and “epidermis development” (GO.0008544). Importantly, the analysis showed a tight relationship of these processes with the “skin development” process (GO.0043588) (**Figure S5**), confirming the high specificity of the mucus exuded proteins. Processes related to “*de novo* post-translational protein folding” (GO.0051084), “protein import into mitochondrial outer membrane” (GO.0045040), “nucleoside diphosphate phosphorylation” (GO.0006165), and “purine ribonucleoside metabolism” (GO.0046128) completed the biological enrichment panel for DESs (**Figure S5**). The full list of the Gene Ontology (GO) biological process enrichment analysis for skin mucus proteome is shown in **Table S5**.

Importantly, at the cellular component level, most of DESs were classified in GO terms as “extracellular region” (GO.0005576), indicating their involvement in exudative processes (**Figure S6**). Other GO identified for DESs were “membrane-bound vesicle” (GO.0031988) and the “melanosome compartment” (GO.0042470), “sarcomere” (GO.0030017), “myofibril” (GO.0030016), and “cell leading edge structures” (GO.0031252) (**Figure S6; Table S6**). Their positional distribution in the proteinteractome showed that the “extracellular region” (GO.0005576) covered almost all the nodes included in the network: 17 DESs (16 up-regulated DESs; one down-regulated DESs) from the 22 possible interacting proteins (**Figure S6**).

**Figure 4:**
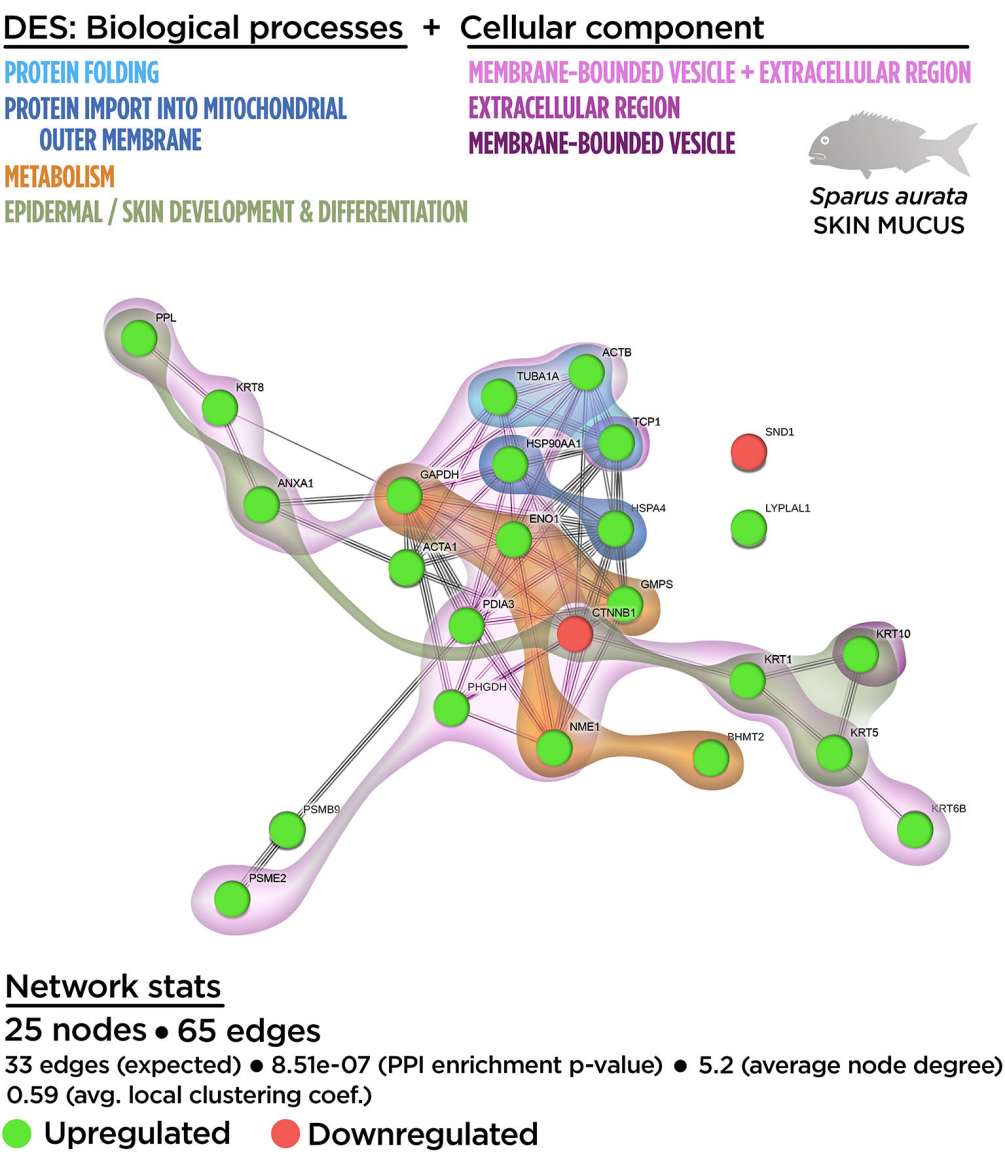
Integrative ameboid graphic for the biological processes and cellular components obtained from the skin proteinteractome analysis in gilthead sea bream fed with SDPP supplemented diet. Each node represents one differential expressed spots (DES) obtained from the skin proteomic analysis. The DES modulatory profile is represented with green (upregulated) and red (downregulated) into each node. The integrative cluster analysis groups the DESs classified into each of the biological processes or cellular components indicated in color (on top). Keratin, Type I Cytoskeletal 10 (KRT10) and T-complex 1 (TCP1) genes are exclusively clustered into membrane-bound vesicle and extracellular region, respectively. The functional network statistics (Network stats) details are indicated (bottom).

Similar to the DEGs, the relationship between the biological processes and cellular component GO terms was evaluated by an integrative cluster analysis (**Figure 4**). The results showed an intimate association between the biological processes (including epidermal and skin development and differentiation) and metabolism (into the context of mucus exudation). Importantly, these data confirms the nature of the skin mucus sample analyzed.

### Skin Mutiomics-Based Interactome Analysis: Merging the Tissue and Exuded Mucus Into a Biological Context of Response

To better understand the skin mucosa functionality on fish fed SDPP, a multi-omics-based interactome analysis performed. This integrative tool was used to merge the transcriptome response determined at tissue level and the proteome profile at the mucosal level in order to interpret data from a holistic perspective. The multinteractomics analysis was constituted by 115 nodes consisting in 93 DEGs (54 up-regulated; 39 down-regulated) and 22 DESs (20 up-regulated; two down-regulated) that in turn were responsible of 313 edges in the functional network. In particular, 88 nodes formed part of the main interaction network: 67 DEGs (43 up-regulated DGEs; 24 down-regulated DGEs) and 21 DESs (19 up-regulated DESs; two down-regulated DESs). This data indicated that our strategy elucidated the integrated context of response, considering expressed genes and synthesized proteins, in fish fed the SDPP diet for 76.9% of the DEGs with gene annotation and 60% of the DESs. On the other hand, 26 DEGs and one DES showed no interaction with the main functional network obtained (**Figure S7**).

As shown in **Figure S7**, the GO enrichment analysis showed several biological processes that were also identified in the transcripteractome (“RNA splicing”; “ribonucleoprotein complex biogenesis”; “intracellular protein transport”; “protein transport”; “protein localization to membrane”; “cellular catabolic process”) and in the proteinteractome (“*de novo* post-translational protein folding”; “skin development”). All of them increased their total number of genes/proteins represented, except in the case of the “RNA splicing” and “ribonucleoprotein complex biogenesis” that showed no variations. Importantly, four new biological processes were identified: “defense response” (GO.0006952), “innate immune response” (GO.0045087), “response to external stimulus” (GO.0009605), and “anatomical structure development” (GO.0048856). The complete list of the GO terms for the biological processes enrichment obtained from the multinteractome analysis is indicated in the **Table S7**.

**Figure 5:**
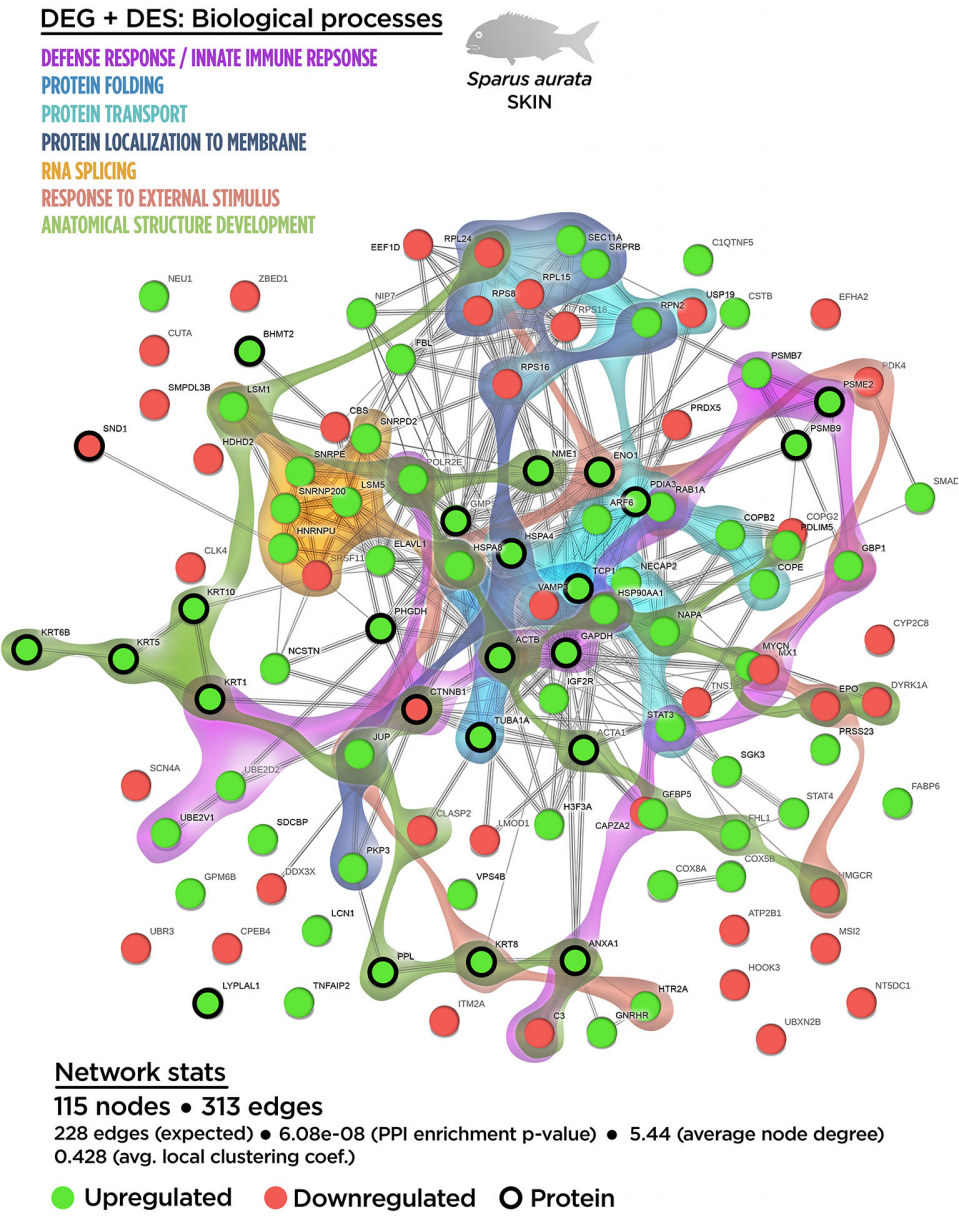
Integrative ameboid graphic for the biological processes obtained for the skin mutiomics-based interactome analysis in gilthead sea bream fed with SDPP supplemented diet. Each node represents one differential expressed gene (DEG) obtained from the skin transcriptomic analysis (circles) or one differential expressed spots (DES) obtained from the skin proteomic analysis (bold circles). The modulatory profile for DEGs or DES is represented with green (upregulated) and red (downregulated) into each node. The integrative cluster analysis groups the DEGs or DESs classified into each of the biological processes indicated in color (on top). The functional network statistics (Network stats) details is also indicated (bottom).

According to the biological process enrichment, the integrative cluster analysis showed no variations compared to the data obtained in the transcripteractome for the interaction between “RNA splicing” and “ribonucleoprotein complex biogenesis” processes (**Figure 5**). The same output than in the transcripteractome was also registered between the “ribonucleoprotein complex” and the “protein transport process”, meanwhile 10 common nodes were determined between “protein transport” and “protein localization to membrane”. The new biological processes identified using multi-omics showed that the distribution of “innate immune response” nodes in the multinteractome was disperse, as well as for the “defense response”, “response to external stimulus” and “anatomical structure development”, indicating a unspecific, but broad *spectrum* of the effects of dietary SDPP on the mucosa (skin and mucus). For instance, a low number of nodes were identified sharing “defense response” with “RNA splicing” (1 node) and “protein transport” (3 nodes). In the case of “response to external stimulus”, none common node was registered with “RNA splicing”, just one node related to “ribonucleoprotein complex biogenesis” and three nodes linked to “protein localization to membrane”. Similarly, the “anatomical structure development” showed two common nodes with “RNA splicing”, three common nodes with “protein transport” and three nodes with “protein localization to membrane”. The “cellular catabolic process” showed a major linkage with “biogenesis” (12 nodes) and “protein transport” (5 nodes) processes, although it was also associated to a minor extend to “defense response” (5 nodes), “response to external stimulus” (2 nodes) and “anatomical structure development” (2 nodes) processes. “*De novo* post-translational protein folding” process was located in the middle of the main core in the multinteractome, merging with the process of “RNA splicing”. In summary, the multinteractome at the level of biological processes showed a clear interaction with low redundancy in the number of nodes that interacted with the above-mentioned processes; thus, suggesting a complementarity biological response at transcriptional and proteome level between those mechanisms modulated in fish fed with SDPP (**Figure 5**).

In the case of the cellular component enrichment analysis, most of the GO terms already identified on the transcriptomic response were also recognized in the multinteractome. The “cell junction structure” (GO.0030054; 17 nodes) detected from the multinteractome celular component enrichment analysis was probably due to the additive effect of the “anchoring junction” (GO.0070161; nine nodes) and “focal adhesion” (GO.0005925; eight nodes) processes (**Figure S8**). No variations in the number of nodes were found for the “spliceosomal-related processes” and the “coated vesicle membrane” compared to the transcripteractome profile. The complete list of the GO terms for the cellular component enrichment from the multinteractome analysis is indicated in the **Table S8**.

**Figure 6:**
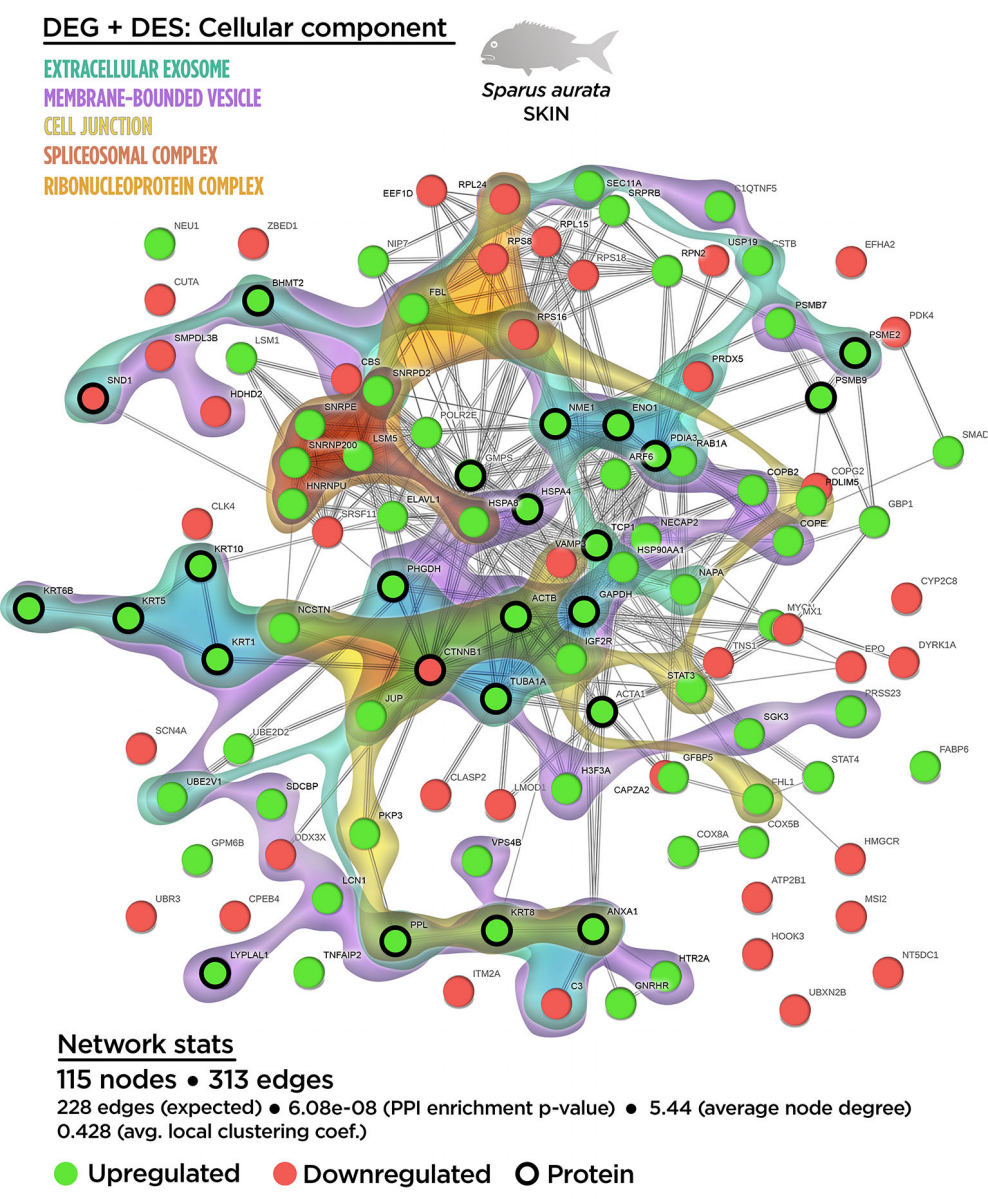
Integrative ameboid graphic for the cellular components obtained for the skin mutiomics-based interactome analysis in gilthead sea bream fed with SDPP supplemented diet. Each node represents one differential expressed gene (DEG) obtained from the skin transcriptomic analysis (circles) or one differential expressed spots (DES) obtained from the skin proteomic analysis (bold circles). The modulatory profile for DEGs or DES is represented with green (upregulated) and red (downregulated) into each node. The integrative cluster analysis groups the DEGs classified into each of the biological processes indicated in color (on top). The functional network statistics (Network stats) details is also indicated (bottom).

**Figure 7:**
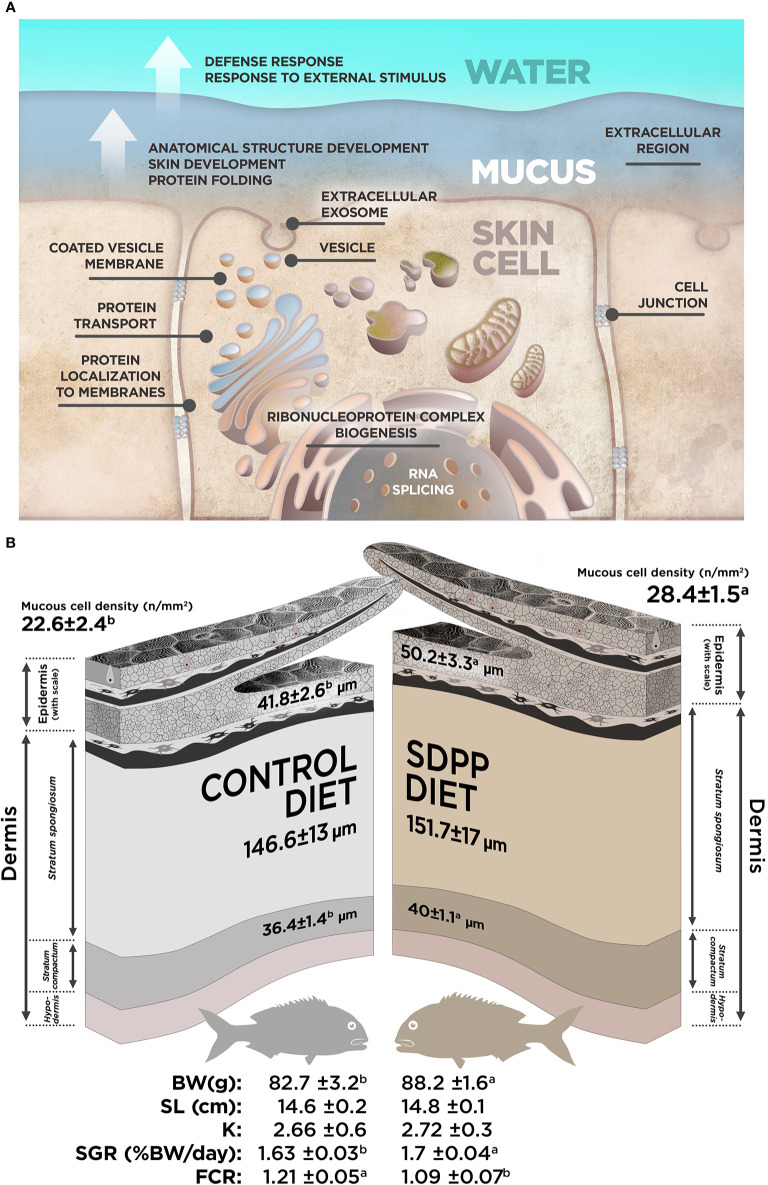
Summary for the data obtained in our study. **(A)** Integrated model of response for the effect of SDPP supplemented diet in gilthead sea bream skin mucosa based on multi-interactome analyses. **(B)** Representation for the most relevant results obtained from the skin histological analysis and somatic growth in gilthead sea bream fed with SDPP supplemented diet.

In agreement with the transcripteractome, the integrative cluster analysis for the cellular component enrichment showed a high relationship with the “spliceosomal complex” (**Figure 6**). The “extracellular exosome” covered most of the interactome; i.e., 36 nodes of the 50 possible (**Figure 6**). Among them, 17 nodes corresponded to DEGs (12 up-regulated DEGs; five down-regulated DEGs). According to the exuded matrix nature of the skin mucus, most of the DESs were included in the “extracellular exosome” with 19 DESs (17 up-regulated DESs; two down-regulated DESs). This result revealed the relevance of an intimate coordination between the transcriptomic and proteomic responses resulting in the exudate of mucus on the surface of the skin’s epithelial tissue in teleost fish. On the other hand, the cell junction structure was also identified in the multinteractome, thus representing 13 nodes from a total of 17 possible (**Figure 6**). Collectively, **Figure 7** represents the summary scheme of global mucosa functionality showing a series of processes promoted and compartments modulated by SDPP, such as RNA splicing (a gene expression-related process), biogenesis, protein transport and localization to membrane, and cell junction structure. Regarding specific mucus properties, an augment also in the modulation of genes and proteins involved in the anatomical structure development of the epithelium, response to external stimulus, and immune defense was also registered in fish fed the SDPP diet.

### Skin Histology and Somatic Growth Performance

**Figure 8:**
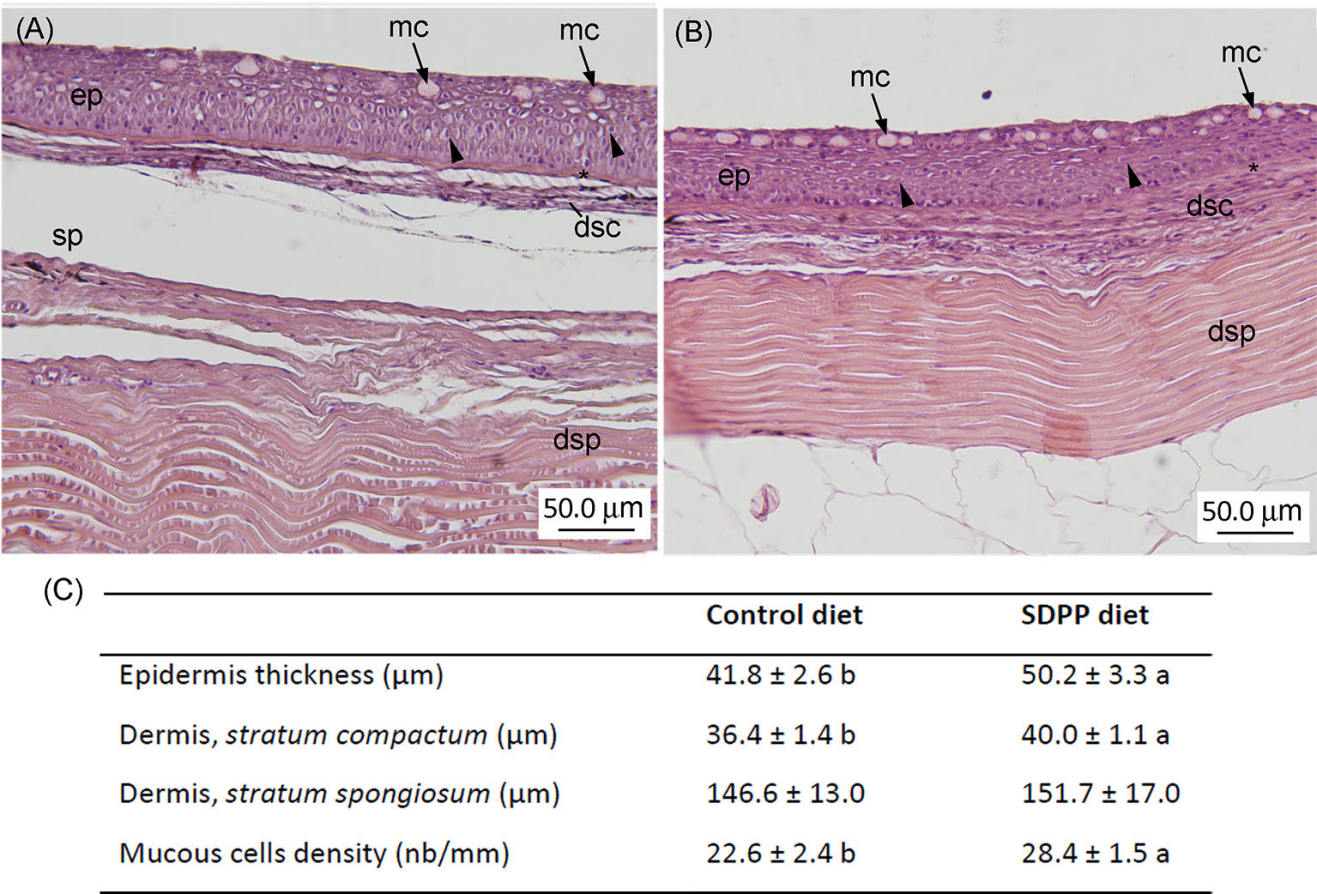
Histological analysis in the skin of gilthead sea bream (*Sparus aurata*) fed diets containing spray-dried porcine plasma (SDPP). Histological organization of the skin in gilthead seabream (*Sparus aurata*) fed **(A)** the control diet and **(B)** the SDPP supplemented diet. **(C)** Histological measurements for epidermis thickness, dermis (*stratum compactum*; *stratum spongiosum)*, and mucous cells density. Different letters within the same row indicate the presence of statistically significant differences between two experimental groups (t-test; *P* < 0.05). Data are expressed as mean ± standard deviation (n = 4).

In order to evaluate whether the molecular response observed on the multi-interactome had consequences on skin anatomy, a histological analysis of this mucosal tissue was conducted. The epidermis from all analyzed fishes had a normal appearance with no visible lymphocytes and macrophages scattered across the stratified squamous epithelium with scattered mucous cells, irrespective to control or SDPP dietary regimes. The skin in both experimental groups had a normal histological organization, being both layers, the epidermis and dermis, clearly differentiated. Importantly, the thickness of the epidermis was higher (p < 0.05) in the animals fed the SDPP diet in comparison to the control group (**Figure 8**). Importantly, this result is consistent with the favoring of the anatomical structure development of the skin obtained from the multi-interactome analysis. Compared to the control diet, the thickness of the *stratum spongiosum* of the dermis was higher in fish fed the SDPP diet (p < 0.05), indicating that this *stratum* is the main target of the SDPP in the skin mucosa. In contrast, no differences in the thickness of the *stratum compactum* were found between both dietary groups (p < 0.05). In addition, the density of epidermal mucous cells, related to mucus production, was higher (p < 0.05) in the SDPP group than in fish fed the control diet.

From a productive point of view, results of growth performance and feed efficiency parameters are summarized in **Table 2**. In particular, BWf and SGR values were 6.2 and 4.1% higher in fish fed the SDPP diet in comparison to the control group (p < 0.05), respectively. No significant differences were found in terms of standard length (SL) and the condition factor K (p > 0.05). In addition, CR values were lower in fish fed the SDPP diet in comparison to those fed the control diet (p < 0.05). Both SGR and FCR were of the major interest parameters on aquaculture production, indicating that the SDPP group grew better and were more efficient in feed use (p < 0.05).

**Table 2:** Somatic growth and feed efficiency in gilthead sea bream (*Sparus aurata*) fed diets containing spray-dried porcine plasma (SDPP).

	Control diet	SDPP diet
BW (g)	82.7 ± 3.2 b	88.2 ± 1.6 a
SL (cm)	14.6 ± 0.2	14.8 ± 0.1
K	2.66 ± 0.6	2.72 ± 0.3
SGR (% BW/day)	1.63 ± 0.03 b	1.70 ± 0.04 a
FCR	1.21 ± 0.05 a	1.09 ± 0.07 b

Different letters within the same row indicate the presence of statistically significant differences between two experimental groups (t-test; p < 0.05). Data are expressed as mean ± standard deviation (n = 4). BW: body weight; SL: standard length; K: Fulton’s condition factor; SGR: specific growth rate; FCR: food conversion ratio.

## Discussion

Organisms interact with the surrounding environment at multiple body sites, including the nasal and oral cavities, the digestive and genitourinary tracts, and the skin surfaces. In contrast to the skin of mammals, fish epidermis is considered a mucosal tissue (13), since it behaves as a mucosal surface that contains associated-lymphoid tissue with abundant mucus-producing cells, lacks keratinization, and harbor living epithelial cells in direct contact with the water medium (12, 49). In the present study, we evaluated the potential benefits of the dietary administration of SDPP in the skin mucosa in a teleost fish model of importance from biological and productive point of views (50). The holistic ‘systems biology’ approach proposed in this current study combines the skin transcriptome and the mucus proteome as a whole response coordinated from the epidermal in its strategic role as outmost-layer barrier. This information, together with skin histology analysis, provides further insights into the teleost skin mucosa functionality.

When included in animal diets, SDPP products resulted in an improvement of somatic growth, feed efficiency parameters, and supported the immune system. It is well known that inclusion levels of 4–8% are recommended for optimal results in higher vertebrates like pigs (25), poultry (24, 26), cats (51) and rats (52). Recently, it was also assayed its beneficial effects on several freshwater and marine fish species of importance due to their economic value such as rainbow trout (30), Nile tilapia (32) and gilthead sea bream (31, 53). Under the present experimental conditions, our results were in agreement with the available literature on different teleost species, confirming the beneficial effects of dietary SDPP inclusion on somatic growth performance and feed efficiency. In addition, we also evidenced that dietary SDPP promoted skin development by increasing the thickness of the epidermis and the *stratum spongiosum* of the dermis, providing new evidence of the dietary supplement at mucosal level. Similarly to the antecedent obtained from intestinal mucosa (53), our study showed that dietary SDPP increased the density of mucous cells in the epidermis. The above-mentioned results on growth, diet utilizationand mucosal tissue development might be attributed to the nutritional profile of SDPP that includes growth factors, immunoglobulins and bioactive peptides (27, 54, 55). Although the benefits of dietary SDPP in livestock and model species are well known (27, 51, 52), there is limited information about the mode of action and mechanistic links of this feed ingredient at mucosal level, especially with regard to the skin-associated lymphoid tissue. Thus, we performed a transcriptomic analysis of the skin by means of SAQ microarrays (39) in order to provide insight into the beneficial effects of SDPP. This analysis was complemented with the proteome analysis of the mucus exuded by epidermal mucous cells using SDS-PAGE (44) with the purpose of determining whether different transcriptomic profiling resulted in different mucous skin proteins. Data from both methodologies were finally integrated into a multi-omics-based interactome analysis in order to provide a holistic approach of the effects of SDPP at the skin level. This strategy allowed providing further insight into the adaptation, responsiveness and trade-offs of the skin at cellular level.

A detailed trancripteractome was built from Biological processes and Cellular components based on GO annotations in order to elucidate how the diet acted on the maintenance of healthy skin mucosal tissue. The transcriptomic analysis revealed that at the cellular component level, DEGs obtained corresponded to the “extracellular exosome” (GO.0070062), “anchoring junction” (GO.0070161) and “focal adhesion” (GO.0005925). These processes might be correlated with the role of skin in chemical and physical protection due to mucus exudation by mucous cells, as well as physical barrier by the enhancement of tight junctions at cell-cell level and/or between the cell and the extracellular matrix. These characteristics favor the protection of the organism in front of the environment fluctuations (12, 15). The effect of SDPP promoting the integrity of mucosal tissues has also been reported in previous studies in mammals where an increase of tight junction molecular markers was observed after the administration of SDPP. However, these studies focused on the intestine and none of them evaluated the modulatory effects of SDPP on the skin (56, 57). Furthermore, no evidences are still available on the underlying cell processes that improved the skin barrier function. In addition, GO results revealed that several biological processes were also up-regulated such as those related to (1) RNA metabolism (“RNA splicing”, GO.0008380; “mRNA processing” GO.0006397; “ribonucleoprotein complex biogenesis”, GO.0022613; and “ribosome biogenesis”, GO.0042254); (2) protein fate to membrane (“protein localization to membrane”, GO.0072657; “membrane budding” (GO.0006900); and “intracellular protein transport”, GO.0006886); (3) “ribonucleoprotein complex” (GO.0030529), and 4) “spliceosomal complex” (GO.0005681). The above-mentioned results based on transcriptomic data demonstrated that SDPP supported the structure and function of the skin by promoting intercellular junctions that provide contact and/or adhesion between neighboring cells or between a cell and the extracellular matrix, conferring strength and adhesiveness to the different layers of the epidermis and dermis (58). The results may be attributed to the nutritional profile of SDPP, rich in proteins and functional peptides (59) that may stimulate cell protein turnover and exudation machinery in mucosal tissues.

One of the most distinctive features of body mucosal tissues is the production and secretion of mucus by goblet and club cells (60). Mucus protects the underlying epithelium from chemical, enzymatic and mechanical damage (61), whereas in the skin it also reduces swimming drag forces (62). In fish, the epidermis is responsible for the production and maintenance of the mucous layer *via* the synthesis and secretion of mucins- the high molecular weight glycoproteins densely coated- which formed a support matrix-web equivalent to the mammalian mucus (15). Soluble proteins, other metabolites and microbiota are trapped in this exuded mucous. The functionality of fish mucus has been deeply studied (15, 63–65). In this way, the mucus proteome has classified proteins as structural, metabolic and protection-related functions (44). In the present study, mucus proteome changed in gilthead seabream fed the diet containing SDPP in comparison to the control group. In the two-dimensional proteome analysis, proteins with different pI or relative molecular mass are identified as separate spots. Such information, together with the quantity (abundance and fold change) and identity of those proteins, allowing us to determine the changes at post-translational modification level. It may also determine the cleaved proteins resulting from mucus proteolytic activity. In our study, the current proteome gel-based approach limited the number of detected DESs to the 300 most abundant proteins. However, changes in the abundance of lower molecular mass presents as mucus soluble proteins can be not discarded.

Beyond the non-specific biological processes determined by the Gene Ontology for the proteinteractome, specific skin mucus proteins have been grouped into relevant functional groups. Thus, two specific groups related to mucus formation have been identified: (1) “cytoskeleton related proteins” (TPM1, TUBA1A, PPL and CTNMA1) previously described as structural mucus related proteins (44, 66); and (2) proteins involved in exocytosis processes and cell motility like ACTA1 and ACTB, which have been extensively reported in the fish mucus proteome (44, 67, 68). Although the function of ANXA10 (member of the calcium and phospholipid binding proteins) in the mucus deserves further investigation, this protein has been associated to exocytosis, as well as differentiation and cellular proliferation processes (69). In addition, ACTR1B was found significantly up-regulated in the skin mucus of fish fed SDPP. This is a conserved protein related to actin and dynactin complex and has been found in different cellular compartments. It has been reported in vesicular structures in the cytoplasm (70) as well as in neutrophil degranulation and in the antigen processing and presentation of exogenous peptide antigens *via* Major Histocompatibility Complex (MHC) class II. Nevertheless, considering their cellular functionality, the above-mentioned proteins may be related to vesicle formation, enhancing the liberation of products from epidermal mucous cells to skin mucus. This data matches with our results obtained from the transcriptome analysis concerning the protein fate. Different fragments of keratin fragments (KRT1, KRT10, KRT36) considered as structural components of the epidermis were differentially expressed in the mucus proteome. Cleaved keratins produced by proteolysis *via* extracellular proteases have been proposed to have putative antimicrobial function as membrane pore-forming peptides in higher vertebrates (71). This is of special relevance since one defence mechanism commonly used by the skin is the production of antimicrobial peptides that can kill invading pathogens and activate the host immune response (15). Furthermore, SDPP also promoted other defensive exuded products as proteins with chaperone activity like HSP70, HSP90, TCP1 and GRP78 (44, 67). Their function is also linked to the immunoproteasome and to the MHC class I pathway, which in turn are related to an activation of innate immune responses. For instance, GRP78 has a potent immunological activity when released from the internal environment of the cell into the extracellular space. Specifically, it feeds anti-inflammatory and pro-resolutory signals in immune networks (72). Most of metabolic-related proteins found up-regulated in the skin mucus have no direct functionality in this protective layer; nonetheless, the resultant products of their enzymatic activity may have an important intracellular signaling functionality (66). Four of the over-expressed proteins were grouped within the cell redox activity, including BHMT, GMPS, PHGDH and PDIA3. BHMT mediates betaine and homocysteine transformation, which is involved in methionine biosynthesis and has been described as a precursor of glutathione biosynthesis (73, 74). GMPS synthetises glutamine, which is the precursor of glutamate, one of the molecules needed in glutathione formation. PHGDH is involved in the serine biosynthesis, which has been described to affect glycine formation in mouse, with an effect on mitochondrial glutathione activity (75). PDIA3 is also involved in cell redox homeostasis (74). The presence of these groups of proteins in skin mucus could be related to an up-regulation of the antioxidant defence system and to a more metabolically active tissue as the upregulation of GMPS indicated (involved in the synthesis of purine nucleotides) (76). In summary, the DESs found in the proteome of skin mucus from fish fed the SDPP diet indicated that this feed ingredient promoted the protective role of mucus, with putatively higher antioxidant and antibacterial properties.

The multi-omics-based interactome was conducted based on transcriptomics and proteomics data and their respective interactomes in order to provide a holistic approach of the effects of dietary SDPP on the skin functionality and integrity (77). As far as we know, this study is the first one conducted on an animal mucosa, and especially the skin. In brief, this integrative tool was used to merge the effects of the diet on the tissue functionality at transcriptome (tissue level) and proteome profile (mucosal matrix level). The resulting multi-interactome was composed of 115 nodes (including 93 DEGs and 22 DESs) with a strong interrelationship in 313 edges and higher enrichment values of protein-protein interactions (PPI). Interestingly, new identified biological processes were highlighted considering the skin and mucus differential expression together. In particular, the “anatomical structure development” process (GO.0048856; including 37 nodes) is related to the progression of an anatomical structure from an initial condition to its mature state (GO Term definition). Although few approaches existed in the literature on interactomes from mucosal tissues, this “anatomical structure development” GO process was also identified in the gene expression profiling of olfactory ensheathing glial cells from the olfactory mucosa (78) that have the ability to promote regeneration in the nervous system (79). Thus, the presence of this new biological process would explain the bigger epidermis and dermis thickness found in the skin of fish fed the SDPP diet, as well as its enhanced physical barrier function. The “response to external stimulus” process (GO.0009605; 22 nodes) was another “new” cluster and demonstrated the collaborative work of skin cells and mucus exuded matrix to work as interface with the surrounding environment. It is also an evidence of their tight regulation in front of an epidermal challenge and/or stressor. Additionally, more relevant was the identification of the biological processes related to “innate immune response” (GO.0045087) and “defense response” (GO.0006952), which did not appear when both transcripteractome and proteinteractome were analyzed separately. The innate immune response registered an intimate relationship with the defence response. In fact, all their nodes were contained on its cluster, indicating that this response process was mostly related to an immunological context. These results are of special relevance since the skin requires both intact structural and immunological barriers to protect the organism from external aggressions including pathogens. In this sense, the efficacy of SDPP in livestock nutrition has been associated to an improved barrier function of the gut mucosa and the modulation of the mucosal immune response (27). However, no previous data on the impact of SDPP on the skin was available. Thus, our study confirms that the beneficial role of SDPP is not only restricted to the intestine but it also affects other mucosal tissues of the organism. The immunological-promoting effects of SDPP might be attributed to the immunoglobulin-rich fraction of plasma (80) although other biological peptides may be involved. The skin multi-interactome suggested that the above-mentioned improvements focused no just in a specific cellular way of action, but they also affected several pathways, including gene expression, biogenesis, vesicle formation, protein transport, and protein localization to membrane, all of them corresponding to classical vertebrate response of innate and non-specific defenses.

## Conclusions

The mucous tissue presents two main matrices: the tissue and the secreted mucus that fulfills a wide range of functions mainly aimed at the protection of the epithelial layer. In the current study, we studied for the first time both matrices together in the skin mucosa using a holistic multi-omics approach. The skin transcripteractome revealed a gene expression profile that favors molecular mechanisms associated to transcriptional processes, biogenesis, vesicle formation, protein transport, and protein localization to membrane, whereas mucus proteome enhanced the protective role of mucus, with putatively higher antioxidant and antibactericidal properties. The multi-interactome analysis (integrating data from skin and mucus) evidenced the interrelationship and synergy between the skin metabolism and the exuded mucus functions, improving tissue development, the increase in the thickness of epidermis and the *stratum spongiosum* of the dermis and goblet cell density, the innate defenses and environment recognition. These responses are sustained on a series of processes and compartments related to protein transport and localization to membrane, and structural support at cell junction level. Additionally, SDPP positively impacted on animal performance, growth and feed utilization.

Thus, the integrative perspective followed in our study shows the stimulation of the cell protein turnover and the activation of the exudation machinery in the skin mucosa. This evidence reflects an intimate crosstalk between skin tissue and its exuded mucus in response to a stimulus. In our study, the utilization of SDPP as dietary supplement and its effect upon the teleost skin as model of study. The multi-omics-based interactome analysis increases the power of detecting true causal genes and regulatory networks and pathways involved, providing a comprehensive understanding of the biological context of response that takes place. According to Suravajhala et al. (77), it may even increase the chance to determine the effect of a variable of interest upon animal health and welfare. Overall, this strategy is applicable for evaluating the effect of any experimental variable on any mucosal tissue functionality, including the wide range of applications this assessment may provide on the studies in the mammalian mucosa.

## Data Availability Statement

The datasets presented in this study can be found in online repositories. The names of the repository/repositories and accession number(s) can be found in the article/**Supplementary Material**.

## Ethics Statement

The animal study was reviewed and approved by IRTA’S committee of ethics and animal experimentation.

## Author Contributions

The conceptualization of the experiment was developed by FER-L, AI, JP, and EG. The methodology carried out was originally proposed by FER-L, AI, and EG. The feeding trial was performed by JF and EG, while sampling was conducted by AI, BO-G, JF, and EG. Microarrays hybridizations, raw data output and processing, and data analysis was made by FER-L and EV-V. The procedure related to proteomic skin exuded mucus, including raw data output and processing, and data analysis was done by AI, BO-G, LF-A, IS, SS-N. FER-L, EV-V, and LP made the conceptualization, methodology and interpretation of the integrative analysis including interactomes, GO terms enrichment, and cluster analysis. The conceptualization and design of figures and tables were in charge of FER-L, AI, BO-G, EV-V, JB, and EG. Funding was obtained by EG. All the authors contributed in the data analysis. FER-L, AI, BO-G, EV-V, and EG wrote the original draft. All authors contributed to the article and approved the submitted version.

## Funding

This study had the financial support of (1) MINECO (project numbers AGL2014-51839-C5-5-R and AGL2015-70637-R); (2) DIETAplus project of the JACUMAR (Junta de Cultivos Marinos, MAPAMA; Spanish government), which is co-funded with FEMP funds (EU); (3) ERC (European Research Council) in MedAID project (Mediterranean Aquaculture Integrated Development; Grant Agreement nb. 727315); and (4) Fondecyt regular (project number 1211841; ANID; Goverment of Chile). EV-V was granted with DYCIT-USACH Postdoctoral fellowship (no. 022043IB). JF has been subsidized by the Industrial PhD program of the Generalitat de Catalunya and TECNOVIT-FARMFAES. Collaboration between Ibero-American researchers has been done under the framework of the network LARVAplus “Strategies for the development and improvement of fish larvae production in Ibero-America” (117RT0521) funded by the Ibero-American Program of Science and Technology for Development (CYTED, Spain). The funder bodies were not involved in the study design, collection, analysis, interpretation of data, the writing of this article or the decision to submit it for publication.

## Conflict of Interest

JP is APC Europe, S.L. employer. FR-L was employed by Consorcio Tecnológico de Sanidad Acuícola, Ictio Biotechnologies S.A.

The remaining authors declare the research was conducted in the absence of any commercial or financial relationships that could be construed as potential conflicts of interest.
