# Supplementary material for: Skin Multi-Omics-Based Interactome Analysis: Integrating the Tissue and Mucus Exuded Layer for a Comprehensive Understanding of the Teleost Mucosa Functionality as Model of Study
Source: Front Immunol. 2021 Feb 4;11:613824. doi: 10.3389/fimmu.2020.613824 (PMC7890662; doi:10.3389/fimmu.2020.613824)
Supplement: Supplementary file 1 [file DataSheet_1.pdf]

Figure S1. Representative 2D-gel showing the differentially expressed spots (DES) in skin mucus. After a cleaning process, the protein extract was separated on 24 cm non-linear pH 3e10 IPG strips, followed by separation using 12.5% SDS-PAGE. Numbers indicate DESs with correspondence with Table S4 identification. Green spots corresponded up-expressed proteins after 90 days fed SDPP diet and red spots corresponded to down-expressed proteins.

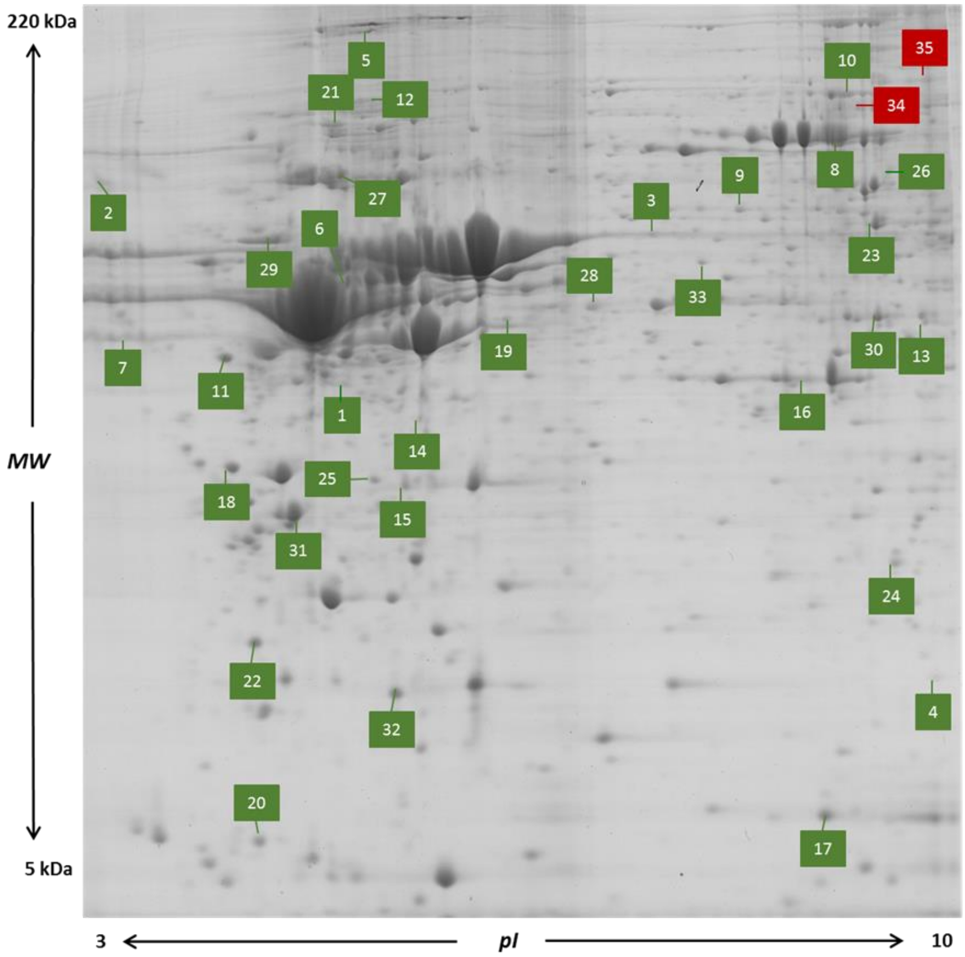

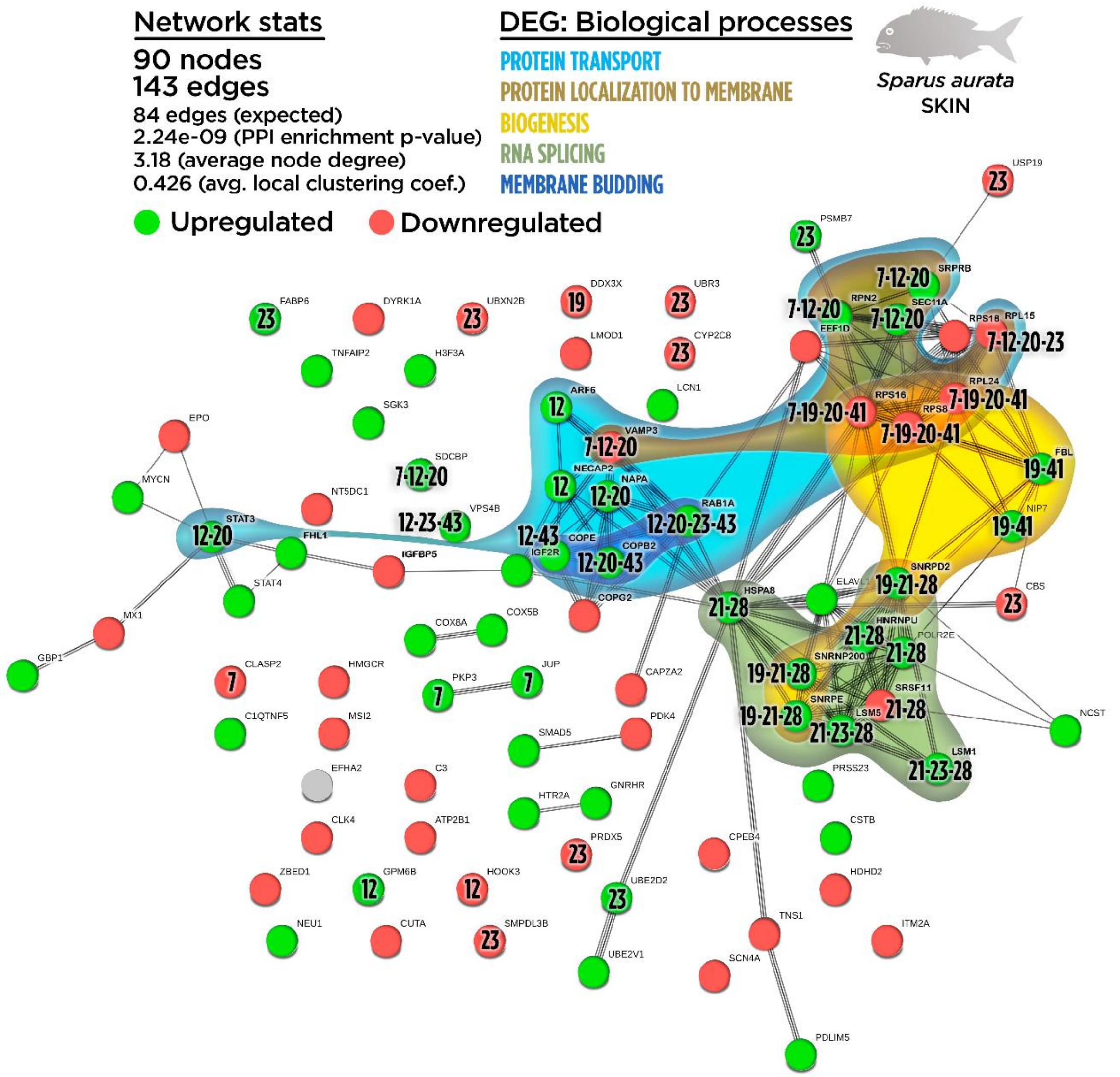

Pathway ID	Pathway description	Observed gene count	False discovery rate
GO.0072657	**7** protein localization to membrane	12	8.77e-05
GO.0015031	**12** protein transport	19	0.000398
GO.0022613	**19** ribonucleoprotein complex biogenesis	9	0.00374
GO.0006886	**20** intracellular protein transport	13	0.00454
GO.0008380	**21** RNA splicing	9	0.00649
GO.0044248	**23** cellular catabolic process	18	0.00735
GO.0006397	**28** mRNA processing	9	0.0198
GO.0042254	**41** ribosome biogenesis	6	0.0436
GO.0006900	**43** membrane budding	4	0.0493

Figure S2. Skin transcripteractome analysis for the biological processes in gilthead sea bream fed with SDPP supplemented diet. The functional network statistics (Network stats) is indicated (upper left). Each node represents one differential expressed gene (DEG) obtained from the skin transcriptomic analysis. The modulatory profile for DEGs is represented with green (upregulated) or red (downregulated) into each node. The number indicated into each node details the pathway

description it belongs. The integrative cluster analysis groups the DEGs classified into each one of the GO biological processes terms indicated in color (upper right). The table shows the set of the GO enrichment pathways represented (bottom).

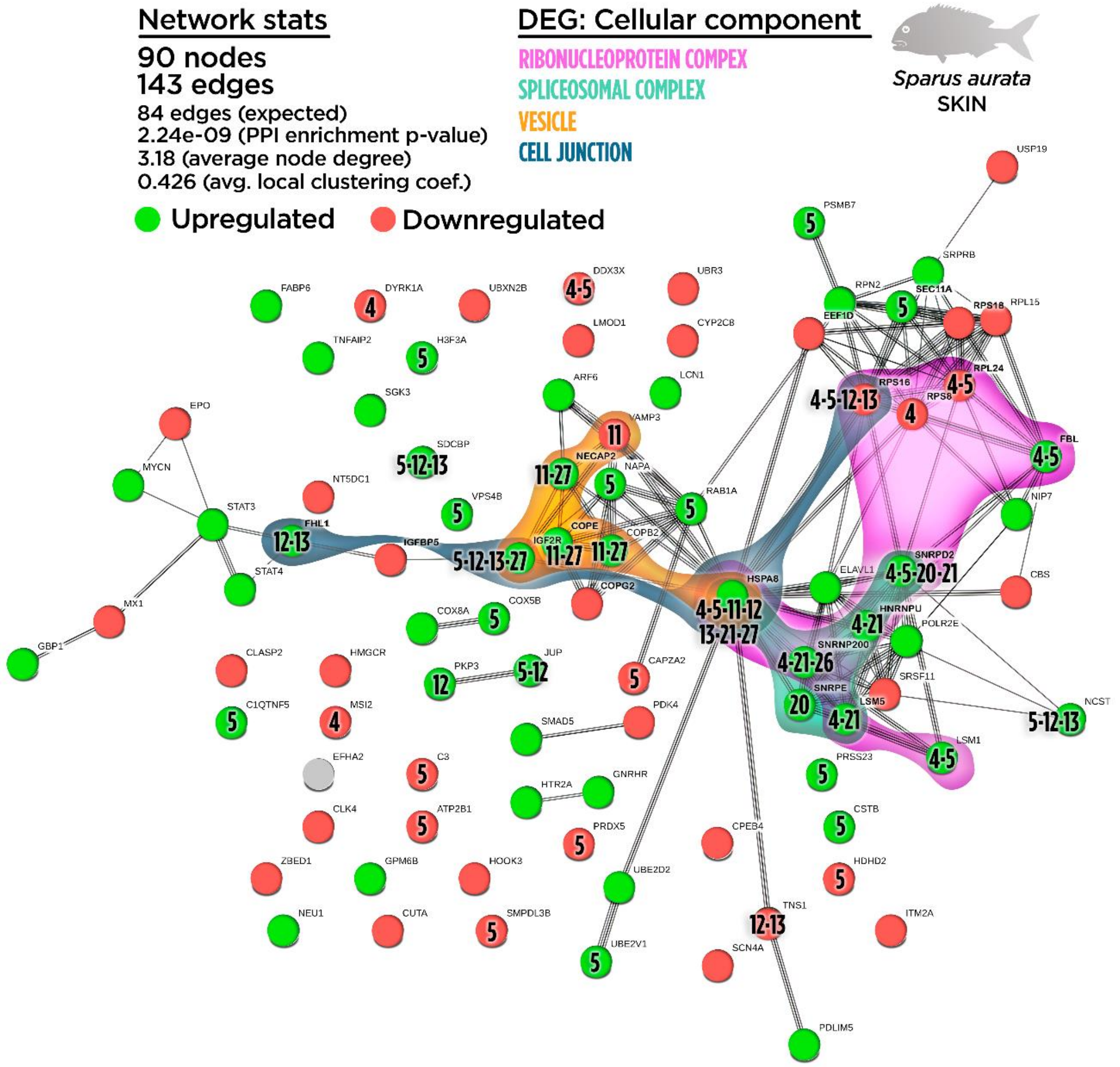

Pathway ID	Pathway description	Observed gene count	False discovery rate
GO.0030529	**4** ribonucleoprotein complex	13	0.00107
GO.0070062	**5** extracellular exosome	28	0.00107
GO.0030662	**11** coated vesicle membrane	5	0.0102
GO.0070161	**12** anchoring junction	9	0.0118
GO.0005925	**13** focal adhesion	8	0.0163
GO.0097525	**20** spliceosomal snRNP complex	3	0.0288
GO.0005681	**21** spliceosomal complex	5	0.0292
GO.0030135	**27** coated vesicle	5	0.0435

Figure S3. Skin transcripteractome analysis for the cellular components in gilthead sea bream fed with SDPP supplemented diet. The functional network statistics (Network stats) is indicated (upper left). Each node represents one differential expressed gene (DEG) obtained from the skin transcriptomic analysis. The modulatory profile for DEGs is represented with green (upregulated) and red (downregulated). The number indicated into each node details the pathway description it

belongs. The integrative cluster analysis groups the DEGs classified into each of the cellular components terms indicated in color (upper right). The table shows the set of the GO enrichment pathways represented (bottom).

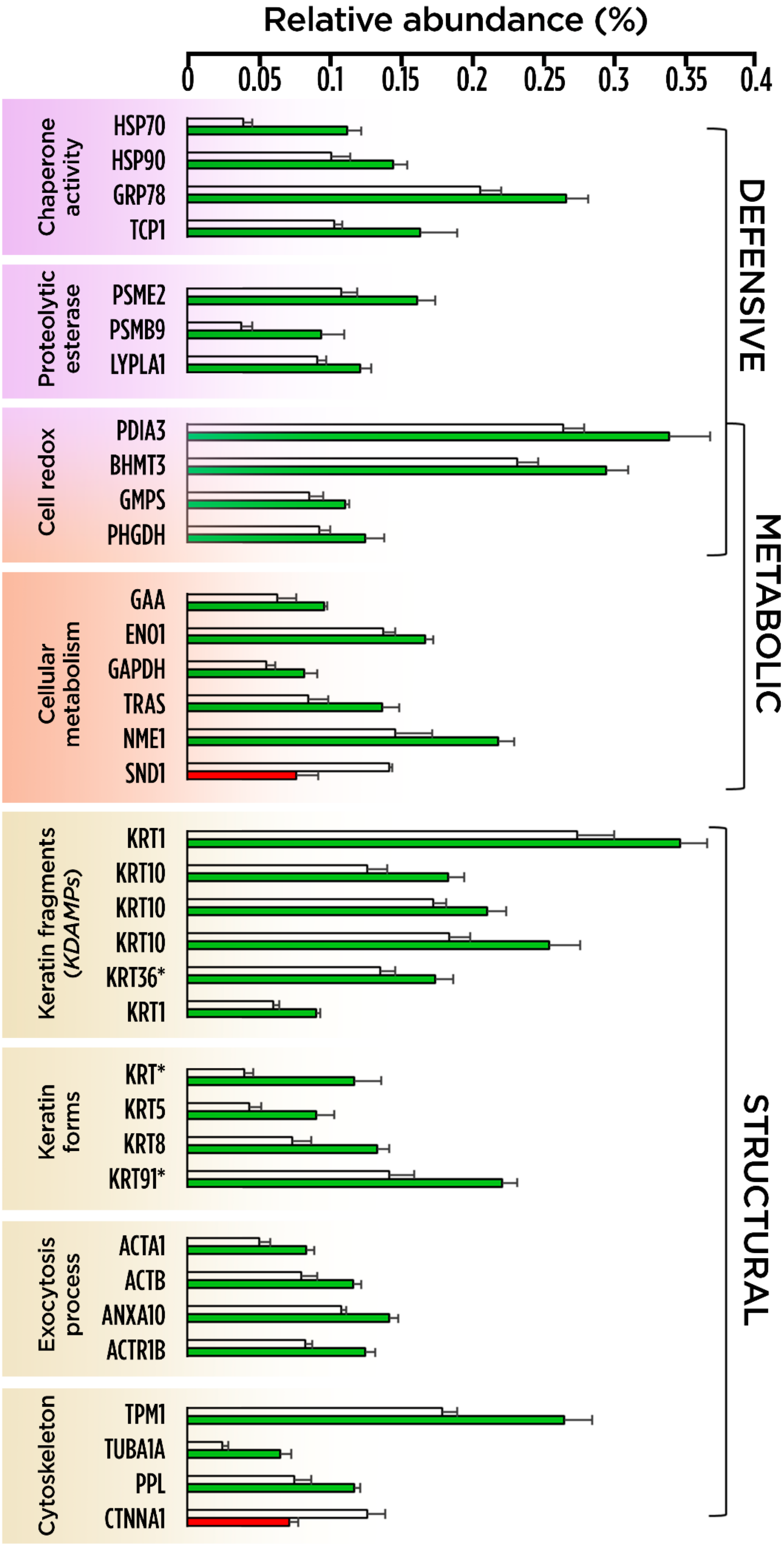

Relative abundance (%)
0
0.05
0.1
0.15
0.2
0.25
0.3
0.35
0.4
Chaperone activity
HSP70
HSP90
GRP78
TCP1
Proteolytic esterase
PSME2
PSMB9
LYPLA1
Cell redox
PDIA3
BHMT3
GMPS
PHGDH
Cellular metabolism
GAA
ENO1
GAPDH
TRAS
NME1
SND1
Keratin fragments (KDAMPs)
KRT1
KRT10
KRT10
KRT10
KRT36*
KRT1
Keratin forms
KRT*
KRT5
KRT8
KRT91*
Exocytosis process
ACTA1
ACTB
ANXA10
ACTR1B
Cytoskeleton
TPM1
TUBA1A
PPL
CTNNA1
DEFENSIVE
METABOLIC
STRUCTURAL

Figure S4. Physiological grouping of differential expressed spots (DESs) in skin mucus. Following their identification, spots were grouped according to main cellular function (indicated on the right column). The relative abundance (in percentage) is indicated for fish fed with control diet (white bars). The modulatory effect for the DES is indicated with green (upregulated) and red bars (downregulated).

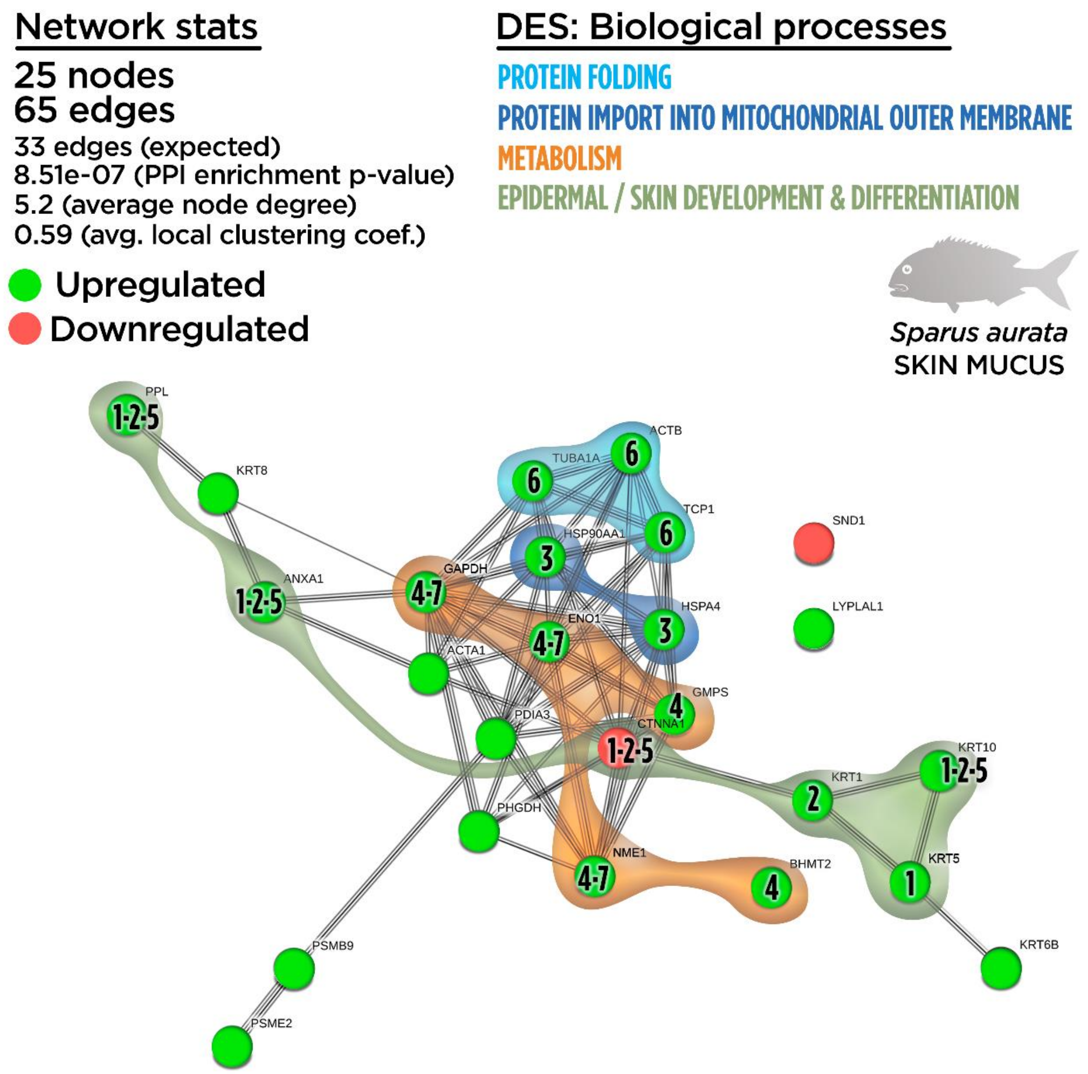

Pathway ID	Pathway description	Observed gene count	False discovery rate
GO.0008544	**1** epidermis development	5	0.0329
GO.0043588	**2** skin development	5	0.0329
GO.0045040	**3** protein import into mitochondrial outer membrane	2	0.0329
GO.0046128	**4** purine ribonucleoside metabolic process	5	0.0329
GO.0009913	**5** epidermal cell differentiation	4	0.0348
GO.0051084	**6** de novo post-translational protein folding	3	0.0348
GO.0006165	**7** nucleoside diphosphate phosphorylation	3	0.0472

Figure S5. Skin proteinteractome analysis for the biological processes in gilthead sea bream fed with SDPP supplemented diet. The functional network statistics (Network stats) is indicated (upper left). Each node represents one differential expressed spot (DES) obtained from the skin proteomic analysis. The modulatory profile for DESs is represented with green (upregulated) or red

(downregulated) into each one. The number indicated into each node details the pathway description it belongs. The integrative cluster analysis groups the DESs classified into each one of the biological processes indicated in color (upper right). The table shows the set of the GO enrichment pathways represented (bottom).

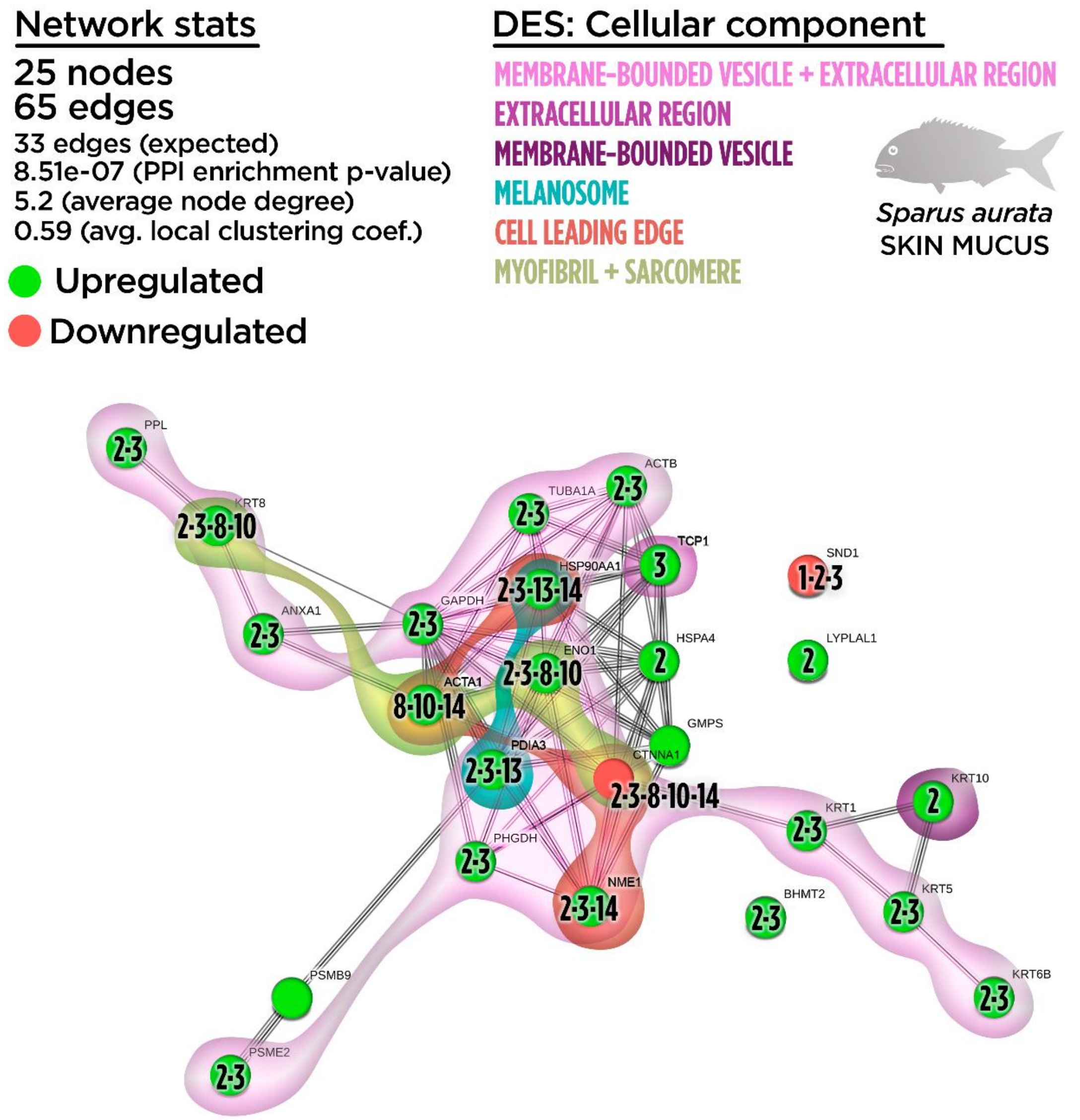

Pathway ID	Pathway description	Observed gene count	False discovery rate
GO.0031988	**2** membrane-bounded vesicle	21	2.73e-11
GO.0005576	**3** extracellular region	22	1.09e-10
GO.0030017	**8** sarcomere	4	0.00732
GO.0030016	**10** myofibril	4	0.00939
GO.0042470	**13** melanosome	3	0.0182
GO.0031252	**14** cell leading edge	4	0.0452

Figure S6. Skin proteinteractome analysis for the cellular components in gilthead sea bream fed with SDPP supplemented diet. The functional network statistics (Network stats) is indicated (upper left). Each node represents one differential expressed spot (DES) obtained from the skin proteomic analysis. The modulatory profile for DESs is represented with green (upregulated) or red

(downregulated) into each node. The number indicated into each node details the pathway description it belongs. The integrative cluster analysis groups those DESs classified into each of the cellular components indicated in color (upper right). The “membrane-bound vesicle + extracellular region” cluster represents the common nodes for both cellular component. Keratin, Type I Cytoskeletal 10 (KRT10) and T-complex 1 (TCP1) genes are exclusively clustered into membrane-bound vesicle and extracellular region, respectively. The table shows the set of GO enrichment pathways represented (bottom).

DEG + DES: Biological processes

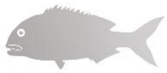

Network stats

115 nodes • 313 edges

228 edges (expected) • 6.08e-08 (PPI enrichment p-value) • 5.44 (average node degree)
0.428 (avg. local clustering coef.)

Upregulated   Downregulated   Protein   47 / 49 Immune-related

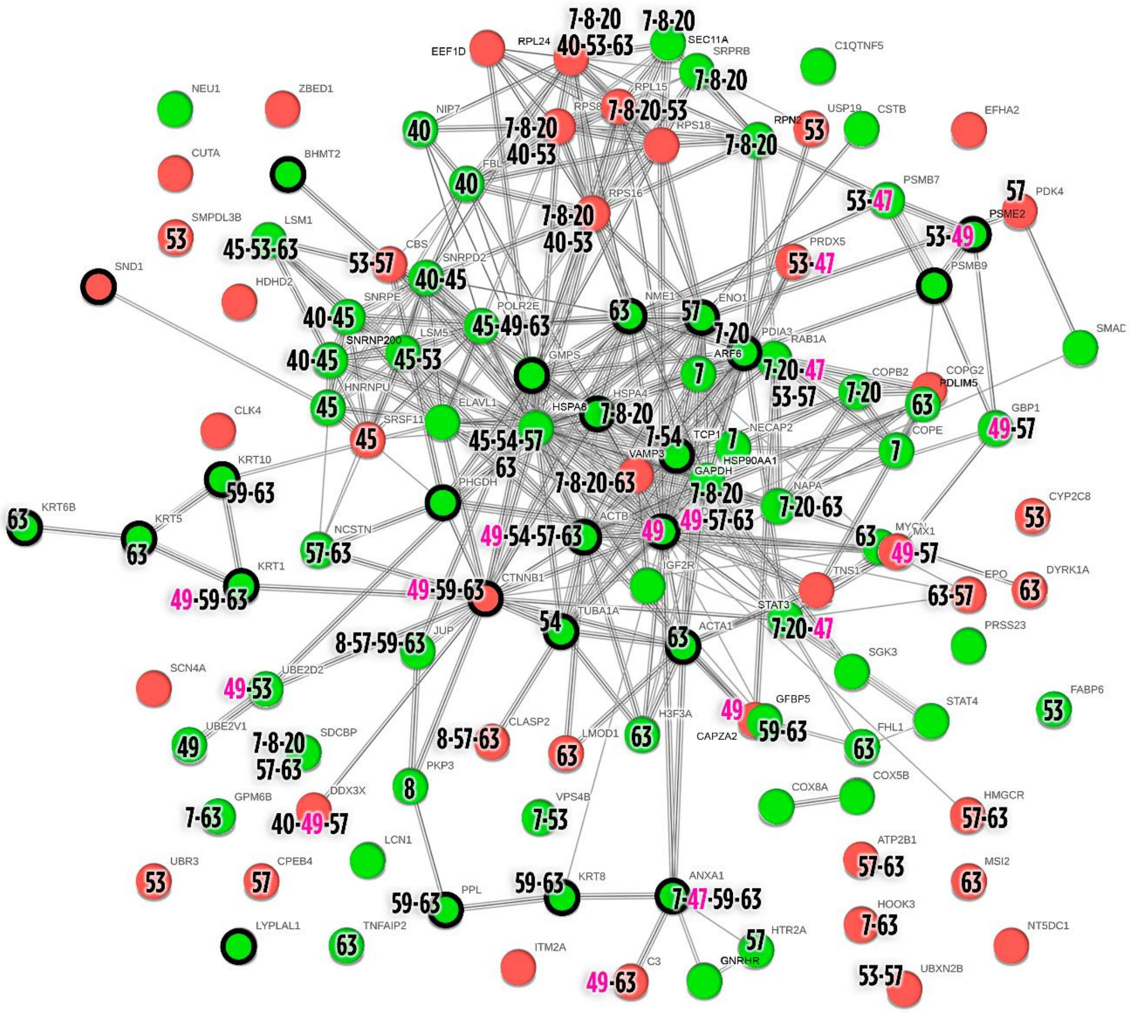

Pathway ID	Pathway description	Observed gene count	False discovery rate
GO.0015031	**7** protein transport	24	1.79e-05
GO.0072657	**8** protein localization to membrane	14	1.79e-05
GO.0006886	**20** intracellular protein transport	16	0.000817
GO.0022613	**40** ribonucleoprotein complex biogenesis	9	0.0133
GO.0008380	**45** RNA splicing	9	0.0226
GO.0006952	**47** defense response	19	0.0245
GO.0045087	**49** innate immune response	15	0.0247
GO.0044248	**53** cellular catabolic process	19	0.0308
GO.0051084	**54** de novo post-translational protein folding	4	0.0317
GO.0009605	**57** response to external stimulus	22	0.0365
GO.0043588	**59** skin development	7	0.0365
GO.0048856	**63** anatomical structure development	37	0.0365

Figure S7. Skin mutiomics-based interactome analysis for the biological processes in gilthead sea bream fed with SDPP supplemented diet. The functional network statistics (Network stats) is indicated (upper left). Each node represents one differential expressed gene (DEG) obtained from the skin transcriptomic analysis (circles) or one differential expressed spot (DES) obtained from the skin proteomic analysis (bold circles). The modulatory profile for DEGs and DESs is represented with green (upregulated) or red (downregulated) into each node. The number indicated into each node details the set of the GO enrichment pathways it belongs and represented in the table (bottom). Since all the genes included in the “innate immune response” (number 49 in the functional network) are also contained in the “defense response” (number 47 in the functional network), all these common nodes are indicated with the number “49”. Those exclusive genes belonging to “defense response” (ANXA1; PRDX5; PMB7; STAT3) are represented in the multinteractome with the number “47”.

DEG + DES: Cellular component

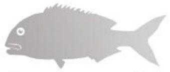

Sparus aurata
SKIN

Network stats

115 nodes • 313 edges

228 edges (expected) • 6.08e-08 (PPI enrichment p-value) • 5.44 (average node degree)
0.428 (avg. local clustering coef.)

● Upregulated ● Downregulated ○ Protein

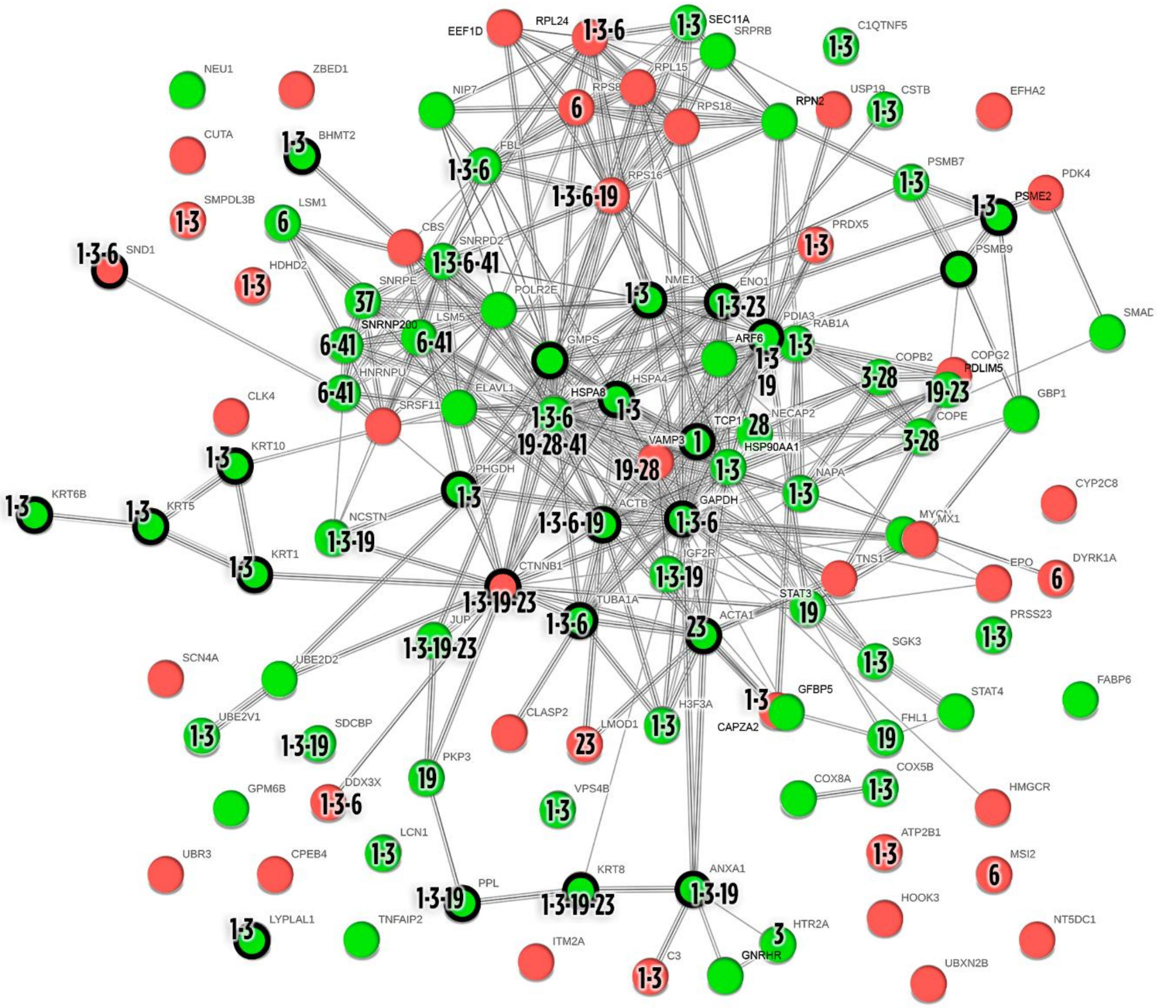

Pathway ID	Pathway description	Observed gene count	False discovery rate
GO.0070062	**1** extracellular exosome	50	2.08e-13
GO.0031988	**3** membrane-bounded vesicle	54	1.18e-12
GO.0030529	**6** ribonucleoprotein complex	17	9.27e-06
GO.0030054	**19** cell junction	17	0.00395
GO.0030016	**23** myofibril	7	0.00685
GO.0030662	**28** coated vesicle membrane	5	0.012
GO.0097525	**37** spliceosomal snRNP complex	3	0.0311
GO.0005681	**41** spliceosomal complex	5	0.0454

Figure S8. Skin mutiomics-based interactome analysis for the cellular components in gilthead sea bream fed with SDPP supplemented diet. The functional network statistics (Network stats) is indicated (upper left). Each node represents one differential expressed gene (DEG) obtained from the skin transcriptomic analysis (circles) or one differential expressed spots (DES) obtained from the skin proteomic analysis (bold circles). The modulatory profile for DEGs or DESs is represented with green (upregulated) and red (downregulated) into each node. The number indicated into each node details the set of the GO enrichment pathways it belongs and represented in the table (bottom).

Table S1. Identification of the 194 differential expressed genes obtained for gilthead sea bream skin mucosa fed with SDPP supplemented diet. FC: fold-change.

Description	Log FC	FC	p value
Lithognathus mormyrus clone lmos7p02c10 mRNA sequence	0.6414713	1.5599192	0.00153371
Unknown	0.52570206	1.439634	0.010551547
Unknown	0.5147443	1.4287409	0.007071802
stress protein HSC701 [Seriola quinqueradiata]	0.49602893	1.4103262	0.049154487
Junction plakoglobin [Salmo salar]	0.49365586	1.4080083	0.014192735
Unknown	0.48652315	1.4010643	0.002614973
small nuclear ribonucleoprotein polypeptide E [Danio rerio]	0.46635658	1.3816159	0.032801934
Unknown	0.4447174	1.3610475	0.048094064
fibrillarin [Danio rerio]	0.44144136	1.3579603	0.03934552
cystatin B [Paralichthys olivaceus]	0.43622443	1.3530587	0.028655587
Unknown	0.43236947	1.3494481	0.031881504
Unknown	0.42346308	1.341143	0.023029305
Unknown	0.42022032	1.3381319	0.033139866
Danio rerio cDNA clone IMAGE:7249010	0.4160576	1.3342764	0.018890742
60S ribosome subunit biogenesis protein NIP7 homolog [Salmo salar]	0.41356376	1.331972	0.019016583
PREDICTED: similar to novel G proteincoupled receptor protein [Danio rerio]	0.412509	1.3309985	0.014803958
Lithognathus mormyrus clone lmos3p01b07 mRNA sequence	0.41180965	1.3303535	0.049357828
Unknown	0.41156697	1.3301297	0.010113281
STAT4 [Tetraodon fluviatilis]	0.40908244	1.327841	0.020372702
Lipocalin precursor [Salmo salar]	0.40146536	1.3208488	0.04062557
fatty acid binding protein H6isoform [Gobionotothen gibberifrons]	0.39823562	1.3178952	0.02238093
Diplodus sargus igfII mRNA for preproinsulingrowth factor II, complete cds	0.3949437	1.3148915	0.023723846
Proteasome subunit beta type 7 precursor [Oncorhynchus mykiss]	0.39178637	1.312017	0.007664731
Unknown	0.38400388	1.3049585	0.035609543
Ribophorin II [Danio rerio]	0.3818583	1.3030192	0.039348535
Complement C1q tumor necrosis factorrelated protein 5 [Salmo salar]	0.37589666	1.2976458	0.029467463
Small nuclear ribonucleoprotein Sm D2 [Oncorhynchus mykiss]	0.35871384	1.2822822	0.04332396
Unknown	0.3560598	1.2799255	0.011128807
plakophilin 3 [Bos taurus]	0.3559757	1.2798508	0.022619555
rCG29738, isoform CRA_b [Rattus norvegicus]	0.35521597	1.2791771	0.03850113
glucocorticoid regulated kinase 3 [Danio rerio]	0.3546758	1.2786982	0.015499827
NECAP2	0.35240585	1.2766879	0.020776788
Neuronal membrane glycoprotein M6b [Salmo salar]	0.34798247	1.2727795	0.02198407
Unknown	0.3457815	1.2708392	0.017526014
PDZ and LIM domain 5 [Xenopus (Silurana) tropicalis]	0.34370893	1.2690148	0.03557238
U6 snRNAassociated Smlike protein LSm5 [Salmo salar]	0.3430843	1.2684655	0.005092626
CXC Motif Chemokine Ligand 1 [Danio rerio]	0.33403754	1.2605362	0.03883394
Unknown	0.3251679	1.2528102	0.01926762
Unknown	0.31890008	1.2473792	0.03410844
Peptidylprolyl isomeraselike 5 [Salmo salar]	0.309557	1.2393271	0.034609944
Unknown	0.3085168	1.2384338	0.015252142
mitochondrial cytochrome C oxidase subunit Vb precursor [Epinephelus coioides]	0.3075003	1.2375616	0.012012399
Four and a half LIM domains protein 1 [Salmo salar]	0.30496052	1.2353848	0.004004599
Unknown	0.30345273	1.2340944	0.039893676
Unknown	0.30144596	1.232379	0.035026606
ELAVlike protein 1 [Salmo salar]	0.3012225	1.2321881	0.018805996
Unknown	0.30099303	1.2319921	0.04588026
Unknown	0.29528716	1.2271292	0.034816783
Coatomer protein complex, subunit beta 2 [Danio rerio]	0.2929508	1.2251436	0.046288684
RAB1, member RAS oncogene family [Mus musculus]	0.2920875	1.2244107	0.03147935
ADP ribosylation factor 79F [Argas monolakensis]	0.28565127	1.2189604	0.04224468

Nicastrin [Danio rerio]	0.27935582	1.2136528	0.047575194
Crystal Structure Of Human UbiquitinConjugating Enzyme Ubch5b Chain B	0.27512363	1.2100978	0.008583799
Gammasecretase subunit PEN2 [Salmo salar]	0.27334884	1.20861	0.03456743
Coatomer subunit epsilon [Oncorhynchus mykiss]	0.27141017	1.206987	0.005996352
DNAdirected RNA polymerases I, II, and III subunit RPABC2 [Esox lucius]	0.26021147	1.1976542	0.003534085
Unknown	0.25243214	1.1912136	0.01455758
Cytochrome c oxidase polypeptide VIIIheart, mitochondrial precursor [Salmo salar]	0.2505296	1.1896437	0.029611208
Lithognathus mormyrus clone lmos8p02h02 mRNA sequence	0.24520712	1.1852629	0.02056361
Unknown	0.24464779	1.1848035	0.006602625
Nmyc downstream regulated 1 isoform 1 [Danio rerio]	0.24240133	1.18296	9.22E-04
Unknown	0.23908947	1.1802475	0.031215368
STAT3 [Tetraodon fluviatilis]	0.23554872	1.1773545	0.02009851
Unknown	0.23488525	1.1768131	0.040468737
alphainduced protein 2 [Salmo salar]	0.2334118	1.1756119	0.04568941
Unknown	0.23220733	1.1746308	0.041350566
rCG26856, isoform CRA_a [Rattus norvegicus]	0.23006794	1.1728902	0.023188604
Histone H3.3 [Oncorhynchus mykiss]	0.22248892	1.1667447	0.020745728
Rasrelated protein Rap1b precursor [Salmo salar]	0.21688437	1.162221	0.033743452
Serine protease 23 [Salmo salar]	0.21559936	1.1611862	0.03737409
Isocitrate dehydrogenase 1 (NADP+), soluble [Danio rerio]	0.20967393	1.1564268	0.021130726
Salmo salar clone HM5_1489 ubiquitinconjugating enzyme E2 variant 1 (ube2v1) mRNA, partial cds	0.20962337	1.1563863	0.020180335
Unknown	0.20435636	1.1521722	0.032343283
Signal peptidase complex subunit 1 [Salmo salar]	0.20145316	1.149856	0.03303562
U6 snRNAassociated Smlike protein LSm1 [Salmo salar]	0.19940156	1.148222	0.01862426
transmembrane protein 134, isoform CRA_d [Mus musculus]	0.19494076	1.1446772	0.032083966
Syntenin1 [Salmo salar]	0.19439557	1.1442447	0.029343855
Morone saxatilis gonadotropinreleasing hormone gene, complete cds	0.18932675	1.1402315	0.003234949
developmentally regulated GTP binding protein 1 [Danio rerio]	0.18771195	1.138956	0.04236321
Hnrnpu protein [Danio rerio]	0.17564076	1.1294659	0.04019753
Unknown	0.1721621	1.1267458	0.041390717
Unknown	0.16864473	1.1240021	0.03534655
Unknown	0.16711475	1.1228107	0.005609962
Unknown	0.1666858	1.1224769	0.020258132
Mothers against decapentaplegic homolog 5 [Salmo salar]	0.16523854	1.1213515	0.026148666
U5 small nuclear ribonucleoprotein 200 kDa helicase [Salmo salar]	0.16397622	1.1203707	0.027312981
vacuolar protein sorting 4b [Danio rerio]	0.15485981	1.1133134	0.048002943
CWC15 homolog [Salmo salar]	0.14393355	1.1049136	0.015938738
Unknown	0.14165954	1.1031734	0.036842406
Lithognathus mormyrus clone lmos9p04f09 mRNA sequence	0.1403956	1.1022073	0.047455817
Signal recognition particle receptor subunit beta [Salmo salar]	0.1157157	1.0835124	0.03348624
Nethylmaleimide sensitive fusion protein attachment protein alpha [Danio rerio]	0.11410403	1.0823027	0.028356358
Signal Sequence Receptor Subunit 2	0.10238664	1.073548	0.032304306
Unknown	-0.082879126	-1.0591296	0.03458261
Unknown	-0.09441037	-1.067629	0.044542737
Unknown	-0.116865374	-1.0843762	0.022153348
Unknown	-0.11785962	-1.0851238	0.03557527
Seriola quinqueradiata RAF1, antiRAF1, SYN genes and PPARG pseudogene	-0.14731067	-1.107503	0.027999554
Lithognathus mormyrus clone lmos7p01H05 mRNA sequence	-0.14895037	-1.1087625	0.007929119
40S ribosomal protein S16 [Salmo salar]	-0.14960247	-1.1092638	0.013540153
Lithognathus mormyrus clone lmos7p05F12 mRNA sequence	-0.15025118	-1.1097627	0.01598806
Splicing factor arginine/serinerich 11 [Salmo salar]	-0.15424255	-1.1128372	0.03273986
5nucleotidase domaincontaining protein 1 [Salmo salar]	-0.1575827	-1.1154166	0.009982088
60S ribosomal protein L15	-0.15774444	-1.1155417	0.049235277
60S ribosomal protein L24	-0.15865467	-1.1162457	0.029612467
Unknown	-0.16892532	-1.1242207	0.040089436
UBX domaincontaining protein 2 [Salmo salar]	-0.17071345	-1.125615	0.014616667

Unknown	-0.171107	-1.1259221	0.026168339
E3 ubiquitinprotein ligase UBR3 [Salmo salar]	-0.18245316	-1.1348119	0.040295906
Protein transport protein Sec24A	-0.1838092	-1.135879	0.03776756
Unknown	-0.18694995	-1.1383545	0.012489211
ribosomal protein S8 [Solea senegalensis]	-0.1886477	-1.1396949	0.043681715
Unknown	-0.19038293	-1.1410666	0.033278722
NMDA receptorregulated protein 1 [Salmo salar]	-0.19312736	-1.1432393	0.037069015
Unknown	-0.1945917	-1.1444002	0.005198306
HMGCoA reductase [Dicentrarchus labrax]	-0.1960394	-1.1455492	0.018828453
Unknown	-0.20589942	-1.1534052	0.024863759
Peroxiredoxin5, mitochondrial precursor [Salmo salar]	-0.21088593	-1.1573987	0.013360743
40S ribosomal protein S18 [Oncorhynchus mykiss]	-0.21157078	-1.1579483	0.04701988
Protein transport protein Sec24A	-0.21350668	-1.1595031	0.014390421
novel protein similar to vertebrate ubiquitin specific peptidase 19 (USP19) [Danio rerio]	-0.21846789	-1.1634973	0.04715364
Unknown	-0.22064476	-1.1652542	0.021923397
Takifugu rubripes erythropoietin (EPO) gene, complete cds, alternatively spliced	-0.22238426	-1.1666601	0.049280334
cytoplasmic linker associated protein 2 [Danio rerio]	-0.22353555	-1.1675915	0.008545361
Unknown	-0.22387356	-1.167865	0.01565747
Unknown	-0.22574493	-1.1693809	0.04658623
Danio rerio ATPase, Ca++ transporting, plasma membrane 1a (atp2b1a), mRNA	-0.22848654	-1.1716052	0.004784739
Response gene to complement 32 protein [Salmo salar]	-0.22872975	-1.1718028	0.037967563
Retinoic acid receptor alpha-B	-0.23052403	-1.173261	0.005737565
Unknown	-0.23856087	-1.1798152	0.003038745
Dual specificity protein kinase CLK4 [Salmo salar]	-0.24269599	-1.1832017	0.017475447
hook homolog 3 (Drosophila), isoform CRA_c [Homo sapiens]	-0.24602742	-1.185937	0.012378187
Unknown	-0.25020674	-1.1893775	0.017804023
Unknown	-0.2510729	-1.1900918	0.021619786
Unknown	-0.25117016	-1.1901721	0.025109254
WW domain containing E3 ubiquitin protein ligase 1 [Xenopus (Silurana) tropicalis]	-0.25392362	-1.1924458	0.032286074
Unknown	-0.25783294	-1.1956813	0.034644138
Unknown	-0.2631263	-1.2000765	0.032616004
Unknown	-0.2654714	-1.2020288	0.0384108
DEAD (AspGluAlaAsp) box polypeptide 21 [Salmo salar]	-0.26908872	-1.2050464	0.006269754
Elongation factor 1delta [Salmo salar]	-0.26909828	-1.2050544	0.04452249
Factincapping protein subunit alpha2 [Salmo salar]	-0.27176207	-1.2072815	0.04309996
Lithognathus mormyrus clone lithmor94 mRNA sequence	-0.2785809	-1.2130011	0.010437972
Unknown	-0.28067237	-1.2147609	0.035229273
Unknown	-0.28295252	-1.2166823	0.039314542
Unknown	-0.2843508	-1.2178621	0.007929849
Unknown	-0.28978688	-1.2224597	0.013783117
Unknown	-0.30683395	-1.2369901	0.006621469
Integral membrane protein 2A [Salmo salar]	-0.3097572	-1.2394991	0.03280156
Unknown	-0.31029797	-1.2399638	0.048538394
Homo sapiens cytoplasmic polyadenylation element binding protein 4 (CPEB4), mRNA	-0.31275088	-1.2420738	0.045925755
voltagegated sodium channel Nav1.4b [Tetraodon nigroviridis]	-0.32990143	-1.2569275	0.006866195
Homo sapiens dualspecificity tyrosine(Y)phosphorylation regulated kinase 1A (DYRK1A) on chromosome 21	-0.34149384	-1.2670679	0.027156198
Unknown	-0.34271628	-1.268142	0.005177283
Unknown	-0.34682697	-1.2717605	0.037584506
Danio rerio insulinlike growth factor binding protein 5 (igfbp5), mRNA	-0.3474216	-1.2722847	0.044099756
muscleblindlike protein 1D [Danio rerio]	-0.35480168	-1.2788098	0.00596948
Nucleolar GTPbinding protein 1 [Salmo salar]	-0.35815242	-1.2817833	0.048030466
PREDICTED: hypothetical protein [Danio rerio]	-0.36838058	-1.290903	0.025063135
Unknown	-0.38919622	-1.3096635	0.020842616
novel protein similar to vertebrate tensin 1 (TNS1) [Danio rerio]	-0.3904924	-1.3108407	0.041675013
Unknown	-0.39972684	-1.3192581	0.035277046
DnaJ (Hsp40) homolog, subfamily B, member 12 [Danio rerio]	-0.4056005	-1.3246402	0.01274196

Unknown	-0.40855235	-1.3273532	0.014583143
Cystathioninebetasynthase a [Danio rerio]	-0.4156055	-1.3338584	0.005819052
Salmo salar clone ssalrgf527146 RNAbinding protein Musashi homolog 2 putative mRNA, complete cds	-0.42039254	-1.3382916	0.024551108
Unknown	-0.42799997	-1.3453672	0.007983315
Unknown	-0.44188645	-1.3583794	0.036319997
cytochrome P450 2N1 [Oryzias latipes]	-0.44328302	-1.359695	0.007702444
Takifugu rubripes Mx (Mx) gene, complete cds	-0.4481115	-1.3642533	0.012160033
Unknown	-0.44843885	-1.3645629	0.010219141
Unknown	-0.4678013	-1.3830001	0.02690945
CutA homolog precursor [Salmo salar]	-0.46812817	-1.3833135	0.014634373
Unknown	-0.47275776	-1.3877597	0.004315861
Lithognathus mormyrus clone lmos7p04d05 mRNA sequence	-0.47949213	-1.3942528	0.028006213
haloacid dehalogenaselike hydrolase domain containing 2 [Danio rerio]	-0.49458694	-1.4089173	0.015133366
Cytochrome c oxidase polypeptide VIIaliver/heart, mitochondrial precursor [Salmo salar]	-0.49711943	-1.4113927	0.014214148
Prkar2aa protein [Danio rerio]	-0.523292	-1.4372311	0.010254437
pyruvate dehydrogenase kinase, isoenzyme 1, isoform CRA_g [Rattus norvegicus]	-0.54318666	-1.4571877	0.0365411
Unknown	-0.55551195	-1.4696901	0.010885481
Unknown	-0.57018447	-1.4847134	0.030403653
PREDICTED: similar to ZBED1 protein [Danio rerio]	-0.61258596	-1.5289974	0.017924473
Unknown	-0.61443686	-1.5309603	0.041099913
Vesicleassociated membrane protein 3 [Salmo salar]	-0.62210745	-1.5391219	0.034277666
HSC71 [Rivulus marmoratus]	-0.6347294	-1.5526465	0.039981253
Unknown	-0.6633558	-1.5837623	0.007013331
Sadenosylmethionine synthetase isoform type1 [Salmo salar]	-0.66609544	-1.5867727	0.025390612
Gamma-2-COP	-0.7075603	-1.6330402	0.021634823
Unknown	-0.7219495	-1.6494094	0.049157176
Efha2 protein [Mus musculus]	-0.73936963	-1.6694462	0.047796432
Unknown	-0.74810785	-1.6795886	0.011837308
Unknown	-0.76574683	-1.7002499	0.01336607
Unknown	-0.8784937	-1.8384548	0.010551265
acid sphingomyelinaselike phosphodiesterase 3B [Bos taurus]	-1.2609043	-2.396459	0.003252534

Table S2. List of the GO biological process enrichment analysis obtained from the microarrays-based transcriptomic analysis for gilthead sea bream skin mucosa fed with SDPP supplemented diet.

#Pathway ID	Pathway description	Observed gene count	False discovery rate
GO.0090150	establishment of protein localization to membrane	12	9.12E-06
GO.0061024	membrane organization	19	1.16E-05
GO.0045184	establishment of protein localization	22	3.54E-05
GO.0034613	cellular protein localization	20	4.89E-05
GO.0044802	single-organism membrane organization	16	4.89E-05
GO.0072657	protein localization to membrane	12	8.77E-05
GO.0033036	macromolecule localization	26	9.77E-05
GO.0008104	protein localization	23	0.000219
GO.0016482	cytoplasmic transport	15	0.00027
GO.1902580	single-organism cellular localization	16	0.000313
GO.0015031	protein transport	19	0.000398
GO.0006614	SRP-dependent cotranslational protein targeting to membrane	7	0.000487
GO.0006612	protein targeting to membrane	8	0.000542
GO.0016071	mRNA metabolic process	13	0.000542
GO.1902582	single-organism intracellular transport	17	0.00184
GO.0071702	organic substance transport	22	0.00231
GO.0000375	RNA splicing, via transesterification reactions	8	0.00264
GO.0022613	ribonucleoprotein complex biogenesis	9	0.00374
GO.0006886	intracellular protein transport	13	0.00454
GO.0008380	RNA splicing	9	0.00649
GO.0033365	protein localization to organelle	11	0.00649
GO.0044248	cellular catabolic process	18	0.00735
GO.0044085	cellular component biogenesis	21	0.0119
GO.0043624	cellular protein complex disassembly	7	0.0133
GO.0006396	RNA processing	12	0.0156
GO.0000398	mRNA splicing, via spliceosome	7	0.016
GO.0006397	mRNA processing	9	0.0198
GO.0022618	ribonucleoprotein complex assembly	6	0.0198
GO.0006605	protein targeting	9	0.022
GO.0046907	intracellular transport	16	0.022
GO.0048523	negative regulation of cellular process	31	0.022
GO.0044265	cellular macromolecule catabolic process	12	0.0249
GO.0048519	negative regulation of biological process	32	0.0332
GO.0000956	nuclear-transcribed mRNA catabolic process	6	0.0341
GO.0051239	regulation of multicellular organismal process	22	0.0341
GO.0002159	desmosome assembly	2	0.035
GO.0072594	establishment of protein localization to organelle	8	0.0359
GO.0001649	osteoblast differentiation	5	0.0435
GO.0034622	cellular macromolecular complex assembly	10	0.0436
GO.0042254	ribosome biogenesis	6	0.0436
GO.0016032	viral process	11	0.0443
GO.0006900	membrane budding	4	0.0493

Table S3. List of the GO cellular component process enrichment analysis obtained from the microarrays-based transcriptomic analysis for gilthead sea bream skin mucosa fed with SDPP supplemented diet.

#Pathway ID	Pathway description	Observed gene count	False discovery rate
GO.0005829	cytosol	38	3.51E-07
GO.0031988	membrane-bounded vesicle	33	0.00101
GO.0030529	ribonucleoprotein complex	13	0.00107
GO.0070062	extracellular exosome	28	0.00107
GO.0032991	macromolecular complex	37	0.0011
GO.0044422	organelle part	52	0.0011
GO.0044446	intracellular organelle part	51	0.00124
GO.0044421	extracellular region part	31	0.00539
GO.0044444	cytoplasmic part	48	0.00539
GO.0030662	coated vesicle membrane	5	0.0102
GO.0070161	anchoring junction	9	0.0118
GO.0005925	focal adhesion	8	0.0163
GO.0043227	membrane-bounded organelle	63	0.0163
GO.0098805	whole membrane	19	0.0163
GO.0098588	bounding membrane of organelle	21	0.0193
GO.0031090	organelle membrane	25	0.0207
GO.0034715	pICln-Sm protein complex	2	0.0207
GO.0005576	extracellular region	32	0.0288
GO.0097525	spliceosomal snRNP complex	3	0.0288
GO.0005681	spliceosomal complex	5	0.0292
GO.0043226	organelle	64	0.0294
GO.0005687	U4 snRNP	2	0.0297
GO.0030120	vesicle coat	3	0.0394
GO.0043229	intracellular organelle	60	0.0423
GO.0034709	methylosome	2	0.043
GO.0030135	coated vesicle	5	0.0435
GO.0042470	melanosome	4	0.0436
GO.0005682	U5 snRNP	2	0.0454

Table S4. Identification of the 35 differentially expressed spots for gilthead sea bream skin mucus fed with SDPP supplemented diet

SPOT	INT %		INT %		INT	Protein	Gene	Theoretical [c]		Observed [c]						
ID[a]	CTRL[b]	SEM	SDPP[b]	SEM	FOLD	Identity[c]	Symbol [d]	MW	pI	MW	pI	UP[c]	Score[c]	SQ[c]	Species[c]	UniProtKB[d]
1	0.040	0.007	0.118	0.019	2.97	Type I keratin-like protein	KRT 1	35.5	5.02	41	3.92	9	252	75.72	*Sparus aurata*	
2	0.039	0.006	0.113	0.009	2.85	Heat shock protein 70	HSP70	70.8	5.36	76	3.00	23	117	45.20	*N. korthausae*	C0LMQ3
3	0.024	0.005	0.065	0.008	2.70	Tubulin alpha chain	TUBA1A	50.1	5.11	59	6.19	17	127	58.22	*Salmo salar*	P13645
4	0.037	0.008	0.094	0.016	2.51	Proteasome beta 9-SU	PSMB9	23.1	7.80	17	8.79	4	91	20.28	*Oplegnathus fasciatus*	A0A146ZRJ4
5	0.043	0.009	0.090	0.013	2.10	Keratin 5	KRT5	58.3	5.55	222	4.00	3	108	23.14	*N. kuhntae*	P04264
6	0.073	0.014	0.133	0.009	1.82	Keratin. type II cytoskeletal 8	KRT8	55.4	5.00	53	3.97	2	134	18.97	*Fundulus heteroclitus*	A0A1A8HT27
7	0.050	0.008	0.084	0.005	1.66	Actin. cytoplasmic 1	ACTA1	41.7	5.48	46	3.07	3	377	76.27	*Fundulus heteroclitus*	A0A146VU59
8	0.085	0.014	0.137	0.012	1.62	Threonyl-tRNA synthetase	TRAS	82.8	7.01	90	8.09	19	101	26.88	*Danio rerio*	A0A146ZJT9
9	0.103	0.006	0.164	0.026	1.59	T-Complex Protein 1 S-Alpha	TCP1	60.4	6.58	67	6.77	19	143	51.43	*Oreochromis niloticus*	G3NMS8
10	0.075	0.012	0.118	0.004	1.57	Periplakin (putative)	PPL	206.7	6.99	114	7.90	10	124	6.31	*Larimichthys crocea*	I3K6A8
11	0.142	0.018	0.221	0.011	1.56	Keratin-91	KRT91	50.0	5.43	43	3.45	10	117	17.17	*Danio rerio*	O60437
12	0.063	0.014	0.096	0.003	1.52	Alpha-1.4 glucan phosphorylase	GAA	100.5	7.17	102	4.01	13	68	18.92	*Oryzias latipes*	
13	0.083	0.006	0.125	0.007	1.52	Beta-centractin	ACTR1B	42.4	7.23	47	8.46	14	413	61.70	*Larimichthys crocea*	P13645
14	0.060	0.004	0.090	0.003	1.51	Keratin. type II cytoskeletal 1	KRT1	66.0	8.12	35	4.23	18	388	47.05		A0A0F8BFK0
15	0.108	0.012	0.162	0.013	1.50	Proteasome act complex S-2	PSME2	27.8	5.36	29	4.17	3	118	11.84	*Oplegnathus fasciatus*	P04264
16	0.055	0.007	0.082	0.009	1.50	Glyceraldehyde-3-P-DH	GAPDH	36.0	6.84	40	7.38	8	387	57.61	*Pagrus major*	
17	0.146	0.026	0.219	0.011	1.49	Nucleoside diphosphate kinase	NME1	17.0		14	7.56	3	498	65.13	*Sparus aurata*	Q90WD9
18	0.179	0.011	0.266	0.019	1.48	Tropomyosin alpha-1 chain	TPM1	42.2	6.93	31	3.46	15	113	30.96	*Larimichthys crocea*	B5APB7
19	0.080	0.011	0.117	0.006	1.46	Beta actin	ACTB	41.8	4.70	47	4.71	16	230	56.27	*Pagrus major*	A0A0F8AT58
20	0.127	0.014	0.184	0.012	1.45	Keratin. type I cytoskeletal 10	KRT10	59.5	5.48	12	3.78	19	120	48.23		P60709
21	0.101	0.013	0.145	0.010	1.43	Heat shock protein 90	HSP90	83.2	5.01	99	3.89	11	246	46.90	*Miichthys miiuy*	P13645
22	0.184	0.015	0.255	0.022	1.39	Keratin. type I cytoskeletal 10	KRT10	59.5		20	3.55	22	144	50.08		I3RWW5
23	0.093	0.008	0.125	0.013	1.35	D-3-phosphoglycerate DH	PHGDH	55.7	6.16	62	8.49	1	307	23.06	*Gasterosteus aculeatus*	P13645
24	0.092	0.006	0.122	0.008	1.33	Lysophospholipase	LYPLA1	25.1	7.50	24	8.18	7	101	41.38	*Dicentrarchus labrax*	G3NN91
25	0.108	0.004	0.142	0.006	1.32	Annexin	ANXA10	35.0	5.74	30	4.04	3	106	16.61	*Takifugu rubripes*	O75608
26	0.085	0.010	0.111	0.003	1.30	GMP synthase	GMPS	78.5	7.23	76	8.34	6	366	67.98	*Larimichthys crocea*	H2URI3
27	0.206	0.015	0.267	0.015	1.29	Glucose-regulated protein 78	GRP78	72.1	5.08	76	3.92	2	629	42.97	*Larimichthys crocea*	A0A0F8BMB0
28	0.136	0.010	0.174	0.013	1.28	Keratin. type I (Fragment)	KRT36	93.3	5.68	49	5.43	1	182	8.68	*Scleropages formosus*	A0A1D8DE67

29	0.264	0.015	0.339	0.029	1.28	Protein disulfide-isomerase	PDIA3	54.8	4.84	61	3.61	6	167	19.35	*Tetraodon nigroviridis*	A0A0P7V0L9
30	0.232	0.015	0.295	0.016	1.27	BHMT	BHMT3	44.1	6.71	48	8.02	13	959	66.50	*Sparus aurata*	Q4RZP6
31	0.274	0.026	0.347	0.019	1.26	Keratin. type II cytoskeletal 1	KRT1	66.0	8.12	27	3.70	9	54	22.05		V9HXV7
32	0.173	0.010	0.211	0.013	1.22	Keratin. type I cytoskeletal 10	KRT10	59.5		17	4.12	19	126	46.54		P04264
33	0.138	0.008	0.167	0.006	1.21	Enolase 1	ENO1	46.9	6.58	52	6.64	15	369	42.13	*Takifugu rubripes*	P13645
34	0.126	0.013	0.071	0.006	0.56	Catenin Alpha 1	CTNNA1	100.4	6.38	102	7.91	20	128	34.95	*Takifugu rubripes*	H2TDQ7
35	0.142	0.002	0.077	0.015	0.54	Staphylococcal nuclease	SND1	102.2	7.58	109	8.50	4	370	29.42	*Oryzias latipes*	

a Spot number attributed to DSP in text and Supplementary Figure 2.

b Mean and standard error of the mean (SEM) for each individual spot from 5 replicate for Control and SDPP conditions (pools of soluble protein extract from 2 or 3 fish).

c Protein identities, theoretical, and observed MW and pI, peptides matched (unique peptides, UP), percentage sequence coverage (SQ) and species identification were supplied by the Mascot Search Results (Matrix science). Further details of search conditions in Material and Methods section.

d Gene symbol and UniprotKB (http://www.uniprot.org) of each protein were obtained from the Genecards database search process.

Table S5. List of the GO biological process enrichment analysis obtained from the 2-dimensional electrophoresis-based proteome analysis for gilthead sea bream skin mucus fed with SDPP supplemented diet.

Pathway ID	Pathway description	Observed gene count	False discovery rate
GO.0008544	epidermis development	5	0.0329
GO.0043588	skin development	5	0.0329
GO.0045040	protein import into mitochondrial outer membrane	2	0.0329
GO.0046128	purine ribonucleoside metabolic process	5	0.0329
GO.0009913	epidermal cell differentiation	4	0.0348
GO.0051084	de novo posttranslational protein folding	3	0.0348
GO.0006165	nucleoside diphosphate phosphorylation	3	0.0472

Table S6. List of the GO cellular component enrichment analysis obtained from the 2-dimensional electrophoresis-based proteome analysis for gilthead sea bream skin mucus fed with SDPP supplemented diet.

#Pathway ID	Pathway description	Observed gene count	False discovery rate
GO.0070062	extracellular exosome	22	1.66E-14
GO.0031988	membrane-bounded vesicle	21	2.73E-11
GO.0005576	extracellular region	22	1.09E-10
GO.0043209	myelin sheath	6	5.10E-06
GO.0045095	keratin filament	5	1.49E-05
GO.0005829	cytosol	15	3.35E-05
GO.0097433	dense body	2	0.00292
GO.0030017	sarcomere	4	0.00732
GO.0005882	intermediate filament	4	0.00808
GO.0030016	myofibril	4	0.00939
GO.0005634	nucleus	17	0.0117
GO.0045111	intermediate filament cytoskeleton	4	0.0153
GO.0042470	melanosome	3	0.0182
GO.0031252	cell leading edge	4	0.0452

Table S7. List of the GO biological process enrichment analysis obtained for multiomics interactome-based analysis in gilthead sea bream skin mucosa fed with SDPP supplemented diet.

#Pathway ID	Pathway description	Observed gene count	False discovery rate
GO.0090150	establishment of protein localization to membrane	14	1.16E-06
GO.0045184	establishment of protein localization	27	2.88E-06
GO.0061024	membrane organization	22	3.00E-06
GO.0034613	cellular protein localization	24	8.90E-06
GO.0008104	protein localization	29	9.60E-06
GO.0033036	macromolecule localization	32	9.60E-06
GO.0015031	protein transport	24	1.79E-05
GO.0072657	protein localization to membrane	14	1.79E-05
GO.1902580	single-organism cellular localization	20	1.79E-05
GO.0044802	single-organism membrane organization	18	2.64E-05
GO.0016482	cytoplasmic transport	18	3.59E-05
GO.0044085	cellular component biogenesis	30	3.59E-05
GO.0022607	cellular component assembly	27	0.000272
GO.1902582	single-organism intracellular transport	21	0.000308
GO.0071702	organic substance transport	27	0.000584
GO.0030154	cell differentiation	36	0.000622
GO.0033365	protein localization to organelle	14	0.000622
GO.0070972	protein localization to endoplasmic reticulum	8	0.000622
GO.0006886	intracellular protein transport	16	0.000817
GO.0032502	developmental process	46	0.000817
GO.0006605	protein targeting	12	0.00145
GO.0006614	SRP-dependent cotranslational protein targeting to membrane	7	0.00145
GO.0034622	cellular macromolecular complex assembly	14	0.00149
GO.0072594	establishment of protein localization to organelle	11	0.00157
GO.0006612	protein targeting to membrane	8	0.00189
GO.0044767	single-organism developmental process	44	0.00298
GO.0046907	intracellular transport	20	0.00448
GO.0048519	negative regulation of biological process	41	0.00448
GO.0016071	mRNA metabolic process	13	0.00453
GO.0044403	symbiosis, encompassing mutualism through parasitism	15	0.00638
GO.0043933	macromolecular complex subunit organization	26	0.00669
GO.0016032	viral process	14	0.0083
GO.0051649	establishment of localization in cell	23	0.0084
GO.0000375	RNA splicing, via transesterification reactions	8	0.00868
GO.0048523	negative regulation of cellular process	38	0.00898
GO.0010033	response to organic substance	28	0.0106
GO.1902578	single-organism localization	32	0.0108
GO.0051051	negative regulation of transport	11	0.0123
GO.0001649	osteoblast differentiation	6	0.0133
GO.0022613	ribonucleoprotein complex biogenesis	9	0.0133
GO.0051641	cellular localization	25	0.014
GO.0042221	response to chemical	36	0.015
GO.0044699	single-organism process	73	0.015
GO.0009056	catabolic process	22	0.0201
GO.0008380	RNA splicing	9	0.0226
GO.0044763	single-organism cellular process	70	0.0226
GO.0006952	defense response	19	0.0245
GO.0051234	establishment of localization	34	0.0245
GO.0045087	innate immune response	15	0.0247
GO.0071822	protein complex subunit organization	19	0.0285
GO.0060341	regulation of cellular localization	17	0.0301
GO.0043624	cellular protein complex disassembly	7	0.0308
GO.0044248	cellular catabolic process	19	0.0308
GO.0051084	de novo posttranslational protein folding	4	0.0317
GO.0000398	mRNA splicing, via spliceosome	7	0.0365
GO.0002159	GO.0002159	2	0.0365
GO.0009605	response to external stimulus	22	0.0365
GO.0022618	ribonucleoprotein complex assembly	6	0.0365
GO.0043588	skin development	7	0.0365
GO.0044765	single-organism transport	29	0.0365
GO.0045040	protein import into mitochondrial outer membrane	2	0.0365

GO.0048468	cell development	20	0.0365
GO.0048856	anatomical structure development	37	0.0365
GO.0051049	regulation of transport	21	0.0365
GO.0044265	cellular macromolecule catabolic process	13	0.0388
GO.1901575	organic substance catabolic process	19	0.0459
GO.0009057	macromolecule catabolic process	14	0.0487
GO.0033032	regulation of myeloid cell apoptotic process	3	0.0487
GO.0051239	regulation of multicellular organismal process	25	0.0487
GO.0006810	transport	32	0.0491

Table S8. List of the GO cellular component enrichment analysis obtained for multiomics interactome-based analysis in gilthead sea bream skin mucosa fed with SDPP supplemented diet.

#Pathway ID	Pathway description	Observed gene count	False discovery rate
GO.0070062	extracellular exosome	50	2.08E-13
GO.0005829	cytosol	53	2.30E-13
GO.0031988	membrane-bounded vesicle	54	1.18E-12
GO.0044421	extracellular region part	53	6.40E-11
GO.0005576	extracellular region	54	1.40E-08
GO.0030529	ribonucleoprotein complex	17	9.27E-06
GO.0032991	macromolecular complex	48	2.45E-05
GO.0043209	myelin sheath	9	3.33E-05
GO.0005925	focal adhesion	12	9.12E-05
GO.0044444	cytoplasmic part	63	9.12E-05
GO.0070161	anchoring junction	13	9.12E-05
GO.0042470	melanosome	7	0.000119
GO.0044422	organelle part	65	0.000119
GO.0044446	intracellular organelle part	63	0.000283
GO.0005634	nucleus	57	0.00117
GO.0043227	membrane-bounded organelle	81	0.00124
GO.0005912	adherens junction	11	0.00141
GO.0030017	sarcomere	7	0.00395
GO.0030054	cell junction	17	0.00395
GO.0098805	whole membrane	23	0.00529
GO.0071664	catenin-TCF7L2 complex	2	0.00547
GO.0043234	protein complex	37	0.00669
GO.0030016	myofibril	7	0.00685
GO.0043232	intracellular non-membrane-bounded organelle	34	0.00899
GO.0045095	keratin filament	5	0.00899
GO.0030057	desmosome	3	0.00972
GO.0031410	cytoplasmic vesicle	16	0.0106
GO.0030662	coated vesicle membrane	5	0.012
GO.0016023	cytoplasmic membrane-bounded vesicle	15	0.0122
GO.0072562	blood microparticle	5	0.0136
GO.0034715	pICln-Sm protein complex	2	0.018
GO.0097433	dense body	2	0.018
GO.0036464	cytoplasmic ribonucleoprotein granule	5	0.0223
GO.0043226	organelle	79	0.0246
GO.0005687	U4 snRNP	2	0.0299
GO.0016342	catenin complex	2	0.0299
GO.0097525	spliceosomal snRNP complex	3	0.0311
GO.0044428	nuclear part	33	0.0336
GO.0043231	intracellular membrane-bounded organelle	70	0.0351
GO.0034709	methylosome	2	0.0439
GO.0005681	spliceosomal complex	5	0.0454
GO.0030120	vesicle coat	3	0.0454

Table S9: List of orthologue H. sapiens Entrez Gene ID for differential expressed genes (DEGs) or differential expressed spots (DESs) in Sparus aurata. The data source (DEG; DES), S. aurata gene annotation, and *H. sapiens* acronym gene name are detailed.

Source	*S. aurata* annotation	*H. sapiens* acronym gene
DEG	insulin-like growth factor II	IGF2R
	Four and a half LIM domains protein 1	FHL1
	Histone H3.3	H3F3A
	ubiquitinconjugating enzyme E2 variant 1 (ube2v1)	UBE2V1
	Lipocalin precursor	LCN1
	Fatty acid binding protein H6-isoform	FABP6
	Cystatin B	CSTB
	Proteasome subunit beta type-7 precursor	PSMB7
	Ubiquitin-Conjugating Enzyme Ubch5b	UBE2D2
	Serine protease 23	PRSS23
	Syntenin-1	SDCBP
	NECAP2	NECAP2
	Coatomer protein complex, subunit beta 2	COPB2
	Ras-related protein Rab-1A	RAB1A
	Coatomer subunit epsilon	COPE
	Nmyc downstream regulated 1 isoform 1	MYCN
	vacuolar protein sorting 4b	VPS4B
	Nethylmaleimide sensitive fusion protein attachment	NAPA
	Signal recognition particle receptor subunit beta	SRPRB
	Junction plakoglobin	JUP
	Complement C1q tumor necrosis factorrelated	C1QTNF5
	plakophilin 3	PKP3
	Ras-related protein Rap1b precursor	RAB1A
	small nuclear ribonucleoprotein polypeptide E	SNRPE
	Small nuclear ribonucleoprotein Sm D2	SNRPD2
	U6 snRNAassociated Smlike protein LSm5	LSM5
	ELAVlike protein 1	ELAVL1
	DNA-directed RNA polymerases I, II, and III subunit	POLR2E
	U6 snRNAassociated Smlike protein LSm1	LSM1
	Heterogeneous nuclear ribonucleoprotein U	HNRNPU
	U5 small nuclear ribonucleoprotein 200 kDa helicase	SNRNP200
	CWC15 homolog	CWC15
	fibrillarin	FBL
	60S ribosome subunit biogenesis protein NIP7	NIP7
	Ribophorin II	RPN2
	Nicastrin	NCSTN
	Signal peptidase complex subunit 1	SEC11A
	Neuronal membrane glycoprotein M6-b	GPM6B
	Tumor necrosis factor alpha-induced protein 2	TNFAIP2
	Mothers against decapentaplegic homolog 5	SMAD5
	gonadotropinreleasing hormone gene, complete cds	GNRHR
	Heat shock cognate protein 70	HSPA8
	Mitochondrial cytochrome C oxidase subunit Vb	COX5B
	Cytochrome c oxidase polypeptide VIIIheart,	COX8A
	STAT4	STAT4
	STAT3	STAT3
	alphainduced protein 2	NEU1
	similar to novel G proteincoupled receptor protein	HTR2A
	Glucocorticoid regulated kinase 3	SGK3
	PDZ and LIM domain 5	PDLIM5
	ADP ribosylation factor 79F	ARF6

	GTP-binding protein 1	GBP1
	40S ribosomal protein S16	RPS16
	Splicing factor arginine/serinerich 11	SRSF11
	5-nucleotidase domain-containing protein 1	NT5DC1
	60S ribosomal protein L15	RPL15
	60S ribosomal protein L24	RPL24
	UBX domaincontaining protein 2	UBXN2B
	E3 ubiquitinprotein ligase UBR3	UBR3
	Ribosomal protein S8	RPS8
	HMG-Co-A reductase	HMGCR
	Peroxiredoxin5, mitochondrial precursor	PRDX5
	40S ribosomal protein S18	RPS18
	Ubiquitin specific peptidase 19 (USP19)	USP19
	Erythropoietin (EPO)	EPO
	Cytoplasmic linker associated protein 2	CLASP2
	Plasma membrane calcium-transporting ATPase 1	ATP2B1
	Response gene to complement 32 protein	C3
	Dual specificity protein kinase CLK4	CLK4
	Hook homolog 3, isoform CRA_c	HOOK3
	DEAD (Asp-Glu-Ala-Asp) box polypeptide 21	DDX3X
	Elongation factor 1 delta	EEF1D
	F-actin-capping protein subunit alpha-2	CAPZA2
	Integral membrane protein 2A	ITM2A
	Cytoplasmic polyadenylation element binding	CPEB4
	Sodium channel protein type 4 subunit alpha	SCN4A
	Dual-specificity tyrosine(Y)-phosphorylation	DYRK1A
	Insulinlike growth factor binding protein 5 (igfbp5)	IGFBP5
	Muscle-blind-like protein 1D	LMOD1
	Novel protein similar to vertebrate tensin 1 (TNS1)	TNS1
	Cystathionine beta-synthase	CBS
	RNA binding protein Musashi homolog 2	MSI2
	Cytochrome P450 2N1	CYP2C8
	Mx (Mx) gene	MX1
	Cut-A homolog precursor	CUTA
	Haloacid dehalogenase-like hydrolase domain	HDHD2
	[Pyruvate dehydrogenase (acetyl-transferring)]	PDK4
	Zinc finger BED domain-containing protein 1	ZBED1
	Vesicle-associated membrane protein 3	VAMP3
	Coatomer subunit gamma-2	COPG2
	EF-hand domain-containing family member A2	MICU3
	Acid sphingomyelinaselike phosphodiesterase 3B	SMPDL3B
	Type I keratin-like protein	KRT6B
	Heat shock protein 70	HSPA4
	Tubulin alpha chain	TUBA1A
	Proteosome beta 9-like subunit	PSMB9
	Keratin 5	KRT5
DES	Keratin, type II cytoskeletal 8	KRT8
	Actin, cytoplasmic 1	ACTA1
	Threonyl-tRNA synthetase	TRAS
	T-complex protein 1 subunit alpha	TCP1
	Periplakin (putative)	PPL
	Keratin-91	KRT1
	Keratin, type II cytoskeletal 1	KRT1
	Proteosome activator complex subunit 2	PSME2
	Glyceraldehyde-3-phosphate dehydrogenase	GAPDH
	Nucleoside diphosphate kinase	NME1

Beta actin	ACTB
Keratin, type I cytoskeletal 10	KRT10
Heat shock protein 90	HSP90AA1
D-3-phosphoglycerate dehydrogenase	PHGDH
Lysophospholipase (esterase activity)	LYPLAL1
Annexin	ANXA1
GMP synthase [glutamine-hydrolyzing]	GMPS
Keratin, type I cuticular Ha6-like (Fragment)	KRT1
Protein disulfide-isomerase	PDIA3
Betaine homocysteine methyltransferase isoform 3	BHMT2
Enolase 1	ENO1
Catenin alpha 1	CTNNB1
Staphylococcal nuclease domain-containing protein 1	SND1
